# EEG-based dynamic emotion recognition using multi-scale wavelet transform with a Spatio-Temporal neural network

**DOI:** 10.1038/s41598-026-53295-9

**Published:** 2026-06-04

**Authors:** R. S. Soundariya, P. Thangaraj

**Affiliations:** 1 Department of Computer Science and Engineering, Bannari Amman Institute of Technology, Sathyamangalam, Tamilnadu India; 2 Department of Computer Science and Engineering, Kangeyam Institute of Technology , Kangeyam, India

**Keywords:** Emotion recognition, EEG signal processing, Multi-scale wavelet transform, Kalman filtering, Reinforcement learning, Deep Q-Networks, Spatio-Temporal convolutional neural networks, Attention mechanism, Computational biology and bioinformatics, Engineering, Mathematics and computing, Neuroscience

## Abstract

Emotion recognition from EEG signals has been one of the most promising areas due to its potential in enhancing human–computer interaction, especially in adaptive systems. This paper proposes a novel emotion recognition system that improves classification accuracy through advanced signal processing, adaptive channel selection, and deep learning techniques. The system starts with the multi-scale wavelet transform to break down EEG signals effectively followed by Kalman filtering with Wavelet denoising that enhances the quality of signal. Emotional peaks are established using Spectral Entropy analysis for accurate epoch determination. For adaptive channel selection, the utilization of Reinforcement based Deep Q-Networks to pick the most informative EEG channels dynamically for each emotional state. In feature extraction, the hybrid model based on Spatio-Temporal Attention Networks (ST-ANs) is adopted to capture spatial and temporal dependencies in the EEG data. Multi-Scale Feature Fusion is utilized to fuse short- and long-term dependencies. Final emotion classification is done through an Ensemble of Graph Neural Networks (GNNs) and Memory-Augmented Neural Networks (MANNs) providing robust adaptability across different subjects and emotional states. The benchmark dataset is collected from kaggle repository such as EEG brainwave, DEAP dataset and Computer game based EEG dataset. The proposed work achieves 98.5% accuracy on the EEG Brainwave Dataset. On the DEAP Dataset, it achieves 94.5% accuracy. For the EDA on Emotion Recognition (S01G1AllChannels), the model reaches 97.6% accuracy, outperforming the existing frameworks in emotion classification.

## Introduction

Recognition of emotions from EEG signals has recently gained much attention for its application in affective computing, human–computer interaction, and in monitoring mental health. Electroencephalography provides a non-invasive way to record brain activity, capturing the neural oscillations that reflect different emotional states. Since emotions are inherently complex and interact between cognitive and physiological responses, EEG is obviously a powerful modality for decoding these states. From EEG measurements of electrical activity in the brain, researchers aim to decode the patterns corresponding to happiness, sadness, anger, or fear. With the specific temporal and frequency characteristics, EEG signals are an ideal candidate for the dynamic characteristics of emotions^1,2^.

Traditional methods for extracting features in emotion recognition with EEG signals mainly depend on the time, frequency, and time–frequency domains. Mean, variance, skewness, and kurtosis are widely used time-domain features to capture the signal’s characteristics. Techniques in the frequency domain, such as Fast Fourier Transform (FFT) and Power Spectral Density (PSD), are used to obtain power features in different frequency bands of EEG, such as delta, theta, alpha, beta, and gamma. Time–frequency techniques such as Wavelet Transform allow for the combined analysis of the temporal and spectral components of the EEG signal for a more complete representation. These handcrafted features are aimed to capture patterns correlated with different emotional states effectively. For classification, traditional machine learning algorithms like Support Vector Machine (SVM), k-Nearest Neighbor (k-NN) and Random Forests(RF) are used to perform the classification. SVM is preferred for its ability to handle high-dimensional feature spaces and binary classification tasks. k-NN offers simplicity and efficiency in small datasets, while RF provide robustness through ensemble learning. These algorithms normally demand extensive feature engineering along with domain expertise, thereby inhibiting the scalability and adaptation to complex large datasets^3,4^.

Deep learning approaches have replaced the extensive manual feature engineering in classification tasks on emotion recognition from EEG signals. Commonly used neural network architectures include Convolutional Neural Networks (CNNs), Recurrent Neural Networks (RNNs), Long Short-Term Memory (LSTM) networks, and Transformers to learn spatial and temporal patterns directly from raw or very lightly processed EEG data. Spatial correlation within EEG channel arrangements can be captured by CNNs. RNNs and LSTMs are great models to capture temporal dependencies within sequential EEG data. Advanced hybrid models combining CNNs and LSTMs further increase the accuracy of classification by considering spatial and temporal features together^5^. Autoencoders and generative adversarial networks are also considered for the purposes of dimensionality reduction and data augmentation, respectively. These methods achieve high performance, scalability, and adaptability and suitable for real-time emotion recognition applications^6,7^.

Despite considerable progress, the current emotion recognition approaches from EEG signals suffer from several challenges. Traditional methods often depend on manual feature extraction, which is time-consuming, subjective, and fails to capture complex patterns in EEG data. Classical machine learning techniques suffer from high-dimensional EEG datasets and fail to generalize well to new subjects due to inter-subject variability. Deep learning approaches have great potential but are heavy computationally and require labeled datasets that are large. Also, deep models may suffer overfitting, especially when considering noisy and limited EEG data. Such constraints highlight the need for more powerful, scalable, and interpretable methods^8,9^. The objective of the proposed work is to build a robust and efficient framework for emotion recognition from EEG signals that overcomes the limitations of previous methods. This includes the use of Multi-Scale Wavelet Transform (MSWT) for accurate signal decomposition, Kalman Filtering with Wavelet Denoising for noise reduction, and Spectral Entropy Analysis for precise epoch detection. It further integrates the advanced techniques such as Reinforcement Learning with Deep Q-Networks (DQN) for adaptive channel selection and Spatio-Temporal Attention Networks (ST-ANs) for enhanced feature extraction. Finally, it combines Graph Neural Networks (GNNs) and Memory-Augmented Neural Networks (MANNs) to achieve high accuracy and reliability in emotion classification^10,11^.

The main contributions of the proposed work are listed below.

- An end-to-end EEG emotion recognition framework that addresses multi-scale signal decomposition, adaptive channel selection, and spatio-temporal graph-based learning to incorporate subject variability and non-stationary emotional outlines in EEG signals.
- Reinforcement Learning with Deep Q-Networks (ARL-DQN) is used to identify the dynamic channel of the most informative EEG channels, across subjects and temporal epochs, thereby decreasing redundancy and improving generalization.
- A hybrid model that integrates Spatio-Temporal Attention Networks (ST-ANs) for feature extraction and an ensemble of Graph Neural Networks (GNNs) and Memory-Augmented Neural Networks (MANNs) for spatial brain connectivity and long-term temporal emotional dependencies, which are not investigated in conventional attention-based or recurrent models.
- Investigations on various EEG emotion datasets validate that the proposed framework achieves greater performance with respect to accuracy and subject generalization compared to existing state-of-the-art methods.

Disparate existing EEG emotion recognition methodologies that focuses signal preprocessing, channel selection, or classification as isolated stages, the proposed work presents a tightly coupled framework, where each module is aimed to complement the others. Unambiguously, adaptive channel selection is directed by reinforcement learning to handle subject-dependent variability, while graph-based spatial modeling and memory-augmented temporal learning jointly capture functional brain connectivity and long-term emotional dynamics. This amalgamation is inspired by the statement that emotional patterns in EEG signals are fundamentally non-stationary and subject-specific, demanding adaptive and context-aware modeling outside static or pipeline-based elucidations. Section II describes the literature review on various existing feature extraction and classification models in emotion recognition. Section III gives the detailed structure of the proposed work. Section IV explains the results obtained by the proposed work and comparative analysis with existing approaches for three different datasets is performed. Section V concludes the research and gives a future direction in EEG emotion recognition.

## Related work

Cai M, et.al (2024) wrote about emotion recognition through EEG signal with the help of a neural network-based algorithm which provides EEG spectral images. It adopts the multi-layered design, where decomposition of signals and extraction of spectral features is performed by wavelet transforms. The advantage of the method is the possibility to convert EEG signals into a more compact representation by spectral images that increase the recognition accuracy of emotions^13^. Khan SA, et.al. (2024) introduce EEG-ConvNet a convolutional neural network that is a subject-dependent emotion recognition has been tested on EEG signals and is designed considering the subject. This is the CNNs based extraction of the spatial information of the EEG signals with specific usage of it in real time. The benefit is capturing complex patterns in EEG information for improving the subject-dependent accuracy^14^. Tan W, et.al (2024) introduces Spectral-Spatial-Sequential Transformer (S3T) Net model for EEG-based emotion recognition. This approach focuses on the integration of the time–frequency analysis model with the neural network. The primary advantage is the ability of S3T-Net to handle multi-dimensional EEG signals efficiently, making it improve emotion recognition capabilities by leveraging both time and frequency domain features^15^. Akhand MA, et.al (2024) discussed an emotion recognition with improved feature maps for EEG signals through partial mutual information. It has partial mutual information for improving feature selection processes. The irrelevant feature noise will reduce, so that the model performs well in EEG emotion recognition^16^.

The article by Houssein EH, et.al (2024) refers to the Coati Optimization Algorithm (COA) to select the best features to recognise emotions based on EEG signals. The algorithm is a hybrid one, Coati Optimization based on feature selection, which enhances the performance of the recognition system. Its strength lies in the fact that it is able to pick the best features in complicated EEG data with enhanced majorities and minimized computation power^17^. The article by Lu S, et.al. (2023) presents a new approach towards emotion recognition based on EEG through an attention generator trained into a pre-trained Convolution Capsule network (CCN). Its methodology is better featured at improving feature extraction, in the sense that, it pays attention to the significant areas of the signals. The benefit is to add the power of an attention mechanism with a capsule network to enhance the approach to spatial hierarchies of EEG data^18^. The implication of Deng X, et.al (2024) suggests a multi-source contrastive learning (hasty) model of robust cross-subject emotion recognition used in EEG data.

The method improves generalization to unseen subjects by using contrastive learning across multiple subjects. It reduces subject dependency by making the emotion recognition model more robust and applicable to a broader population^19^. Gunda NK, et.al (2024) investigates the lightweight attention mechanisms for the EEG emotion recognition in Brain-Computer Interface (BCI). The proposed model operates with minimal resources while offering accuracy in emotion recognition. The advantage is that this supports real-time BCI applications with efficient resource usage^20^.

Zhang X, et.al (2024) discusses the optimality of dynamics in selection of channels of emotion recognition using EEG recordings. At run time, this method is conducted to choose the most informative EEG channels and has revealed itself to be beneficial in enhancing model performance. It has its merit in the fact that it has enhanced the efficiency of such a model as it minimizes the channels required without compromising the accuracy of the model^21^. In the article by Kouka N, et.al (2023), the authors argued about an EEG Channel Selection and used Binary Particle Swarm Optimization (BPSO) and Recurrent Convolutional Autoencoder (RCA) to Emotion Recognition.It combines EEG channel selection with binary particle swarm optimization and a recurrent convolutional autoencoder for emotion recognition. It efficiently selects relevant EEG channels and performs feature extraction. The advantage is the reduction of dimensionality while enhancing classification performance by focusing on the most relevant data^22^. Wang Y, et.al (2024) discussed a generalized model termed as Mutual Information-based EEG (MI-EEG). It is an emotion recognition system proposed using mutual information without adversarial training. The benefit is the simplicity while ensuring effectiveness in modeling both the relationship between EEG and emotional states without involving setups that are complex with respect to adversarial training^23^. Saha P, et.al. (2024) presents an EEG and electrocardiogram-based multimodal emotion detection system. The strength in this approach is that by using multiple physiological signals for emotion detection, it tends to enhance the robustness as well as accuracy of detection^24^.

Liu S, et.al. (2024) utilizes a Domain-Adaptive Capsule Network (DA-CapsNet) that is a multi-branch capsule network that applies adverse domain adaptation in cross-subject EEG emotion recognition. The benefit is cross-subject variability due to the use of adversarial domain adaptation to enhance the performance across different subjects^25^. Shi X, et.al. (2024) takes cross-subject EEG-based emotion recognition to a more advanced level with multi-source manifold metric transfer learning. Variability among subjects can be taken care of by transfer learning among different sources^26^. Bagherzadeh S, et.al. (2024) presents a subject-independent portable emotion recognition system using synchro squeezing wavelet transform maps of EEG signals and ResNet-18. It provides a portable, real-time emotion recognition system that can be used for various applications inlcuding mental health monitoring^27^. Yang L, et.al (2024) proposed a hierarchical Bayesian spectral regression framework for EEG-based emotion recognition. This method employs hierarchical Bayesian modeling to better model the uncertainty in EEG data to improve the accuracy of emotion recognition. It is robust to noisy and uncertain data^28^.

Lin X, et.al (2023) applies an improved graph neural network with channel selection for EEG emotion recognition. The methodology improves emotion classification by selecting the optimal EEG channels. Its advantage is that it can automatically select informative channels, which improve the recognition accuracy and reduce the computational load^29^. Dwivedi AK, et.al (2024) proposed an Adaptive Flexible Analytic Wavelet Transform for EEG-based Emotion Recognition. This improved time–frequency resolution of the EEG signals will better perceive emotional states. The advantage includes adaptive selection of wavelet functions to improve emotion recognition in dynamic EEG data^30^. Choo S, et.al (2023) proposed a multi-task deep learning framework for EEG-based emotion and context recognition. This method simultaneously recognizes the states of emotion and context through EEG. It has the capacity to perform multitask in order to get the information in contextual levels for improved and accurate emotion detection^31^. Lu K, et.al. (2024) suggested that Convolution-Multilayer Perceptron Network (CMPN) should be used in EEG-Based Emotion Recognition. The benefits of the method are that it can include both spatial and time characteristics of EEG data^32^. Recent investigation in EEG-based emotion recognition, has progressively concentrated in refining robustness and generalization across subjects and recording conditions. Uncertainty-aware domain adaptation methods such as UA-DAAN^33^ and WDANet^34^ report inter-subject distribution shifts by bring into line with latent representations while preserving the model’s predictive uncertainty.

Comparative Analysis of EEG-Based Emotion Recognition Techniques are in Table 1.

**Table 1:** Comparative analysis of EEG-based emotion recognition techniques.

Citation No	Paper title	EEG signal processing technique	Dataset	Model type	Optimization	Cross-subject	Multi-modal	Channel/feature selection
^13^	Cai et al. (2024)	Spectral Image via EEG-Swin Transformer (SWTN)	✓	EEG-SWTNs	✗	✗	✗	✓
^14^	Khan et al. (2024)	Convolutional Networks	✗	CNN	✗	✓	✗	✗
^15^	Tan et al. (2024)	EEG Signals	✗	S3T-Net	✗	✗	✗	✗
^16^	Akhand et al. (2024)	Feature Map Enhancement	✗	Mutual Information	✓	✗	✗	✓
^17^	Houssein et al. (2024)	Coati Optimization	✗	Coati Optimization	✓	✗	✓	✗
^18^	Liu et al. (2023)	Attention Mechanism	✗	CNN + Capsule Network	✓	✓	✓	✓
^19^	Deng et al. (2024)	Multi-Source Contrastive Learning	✗	Contrastive Learning	✓	✓	✗	✗
^20^	Gunda et al. (2024)	Lightweight Attention	✗	CNN + Attention	✓	✓	✓	✓
^21^	Zhang et al. (2024)	Dynamic Channel Selection	✗	CNN	✓	✗	✓	✗
^22^	Kouka et al. (2023)	Binary Particle Swarm Optimization	✗	CNN + Recurrent Autoencoder	✓	✓	✓	✗
^23^	Wang et al. (2024)	Mutual Information	✗	Generalized Model	✗	✓	✗	✗
^24^	Saha et al. (2024)	Multi-modal	EEG + ECG	✗	✗	✓	✗	✗
^25^	Liu et al. (2024)	Adversarial Domain Adaptation	✗	Multi-Branch Capsule Network	✓	✓	✗	✓
^26^	Shi et al. (2024)	Multi-Source Manifold Metric Learning	✗	Metric Transfer Learning	✓	✗	✗	✗
^27^	Bagherzadeh et al. (2024)	Synchrosqueezing Wavelet Transform	✗	ResNet-18	✗	✓	✗	✗
^28^	Yang et al. (2024)	Bayesian Spectral Regression	✗	Bayesian Framework	✗	✗	✗	
^29^	Lin et al. (2023)	Improved Graph Neural Network	✗	GNN	✓	✓	✓	✗
^30^	Dwivedi et al. (2024)	Adaptive Flexible Analytic Wavelet Transform	✗	Wavelet Transform	✗	✓	✗	✗
^31^	Choo et al. (2023)	Multi-task Learning	✗	Deep Learning	✓	✓	✓	✓
^32^	Lu et al. (2024)	Convolution-Multilayer Perceptron	✗	CMLP-Net	✗	✓	✗	✗
^33^	J. Shen et al. (2025)	Uncertainty-Aware Adversarial Domain Adaptation (UA—DAAN)	✓	UA—DAAN	✓	✓	✗	✗
^34^	J. Shen et al. (2025)	Wasserstein-Based Distribution Alignment (WDANet)	✓	WDANet	✓	✓	✗	✗

From the comparative analysis, there are glaring gaps in the research concerning EEG-based emotion recognition. While several sophisticated techniques such as attention mechanisms, mutual information and optimization strategies have been applied. Although the recent articles have investigated several approaches such as cross-subject emotion recognition with domain adaptation, adversarial learning, and meta-learning contexts to diminish inter-subject variability, these approaches result in increasing the complexity of the model as well as variability in training. In order to overcome the issues related to subject instability, the proposed framework introduces adaptive channel selection and spatial–temporal modeling.A multi-modal approach is underrepresented with few studies incorporating ECG in addition to EEG. Additionally, cross-subject generalization remains an under-represented area of study since many models show difficulties in generalizing to unseen subjects^35–39^. Channel and feature selection was highly inconsistent between these studies. Indeed, one of the reasons lightweight and scalable solutions are relevant for real-time applications in brain-computer interfaces.

## Proposed work

The proposed methodologies efficiently bridge the identified research gaps in EEG-based emotion recognition. MSWT offers powerful signal decomposition with rich information in detailed frequency, important for multi-modal integration. Kalman Filtering with Wavelet Denoising reduces noise for enhanced signal clarity, and improves model generalization over subjects. Spectral Entropy Analysis helps to accurately detect epochs and optimize temporal segmentation to represent features more effectively. Reinforcement Learning with Deep Q-Networks (DQN) adaptively selects optimal channels, addressing computational inefficiencies and ensuring scalability. Spatio-Temporal Attention Networks (ST-ANs) extract contextual features by integrating spatial and temporal dimensions, enhancing classification accuracy in multi-modal systems. Finally, the ensemble of Graph Neural Networks (GNNs) and Memory-Augmented Neural Networks (MANNs) leverages graph-based relationships and memory mechanisms for comprehensive emotion classification, improving cross-subject adaptability and real-time applicability in BCIs.

### Multi-scale wavelet transform (MSWT) for signal decomposition

MSWT is one of the powerful signal processing techniques for analyzing signals at multiple resolution levels. This is made possible through wavelet decomposition into a set of coefficients, which in turn are found to represent localized functions in both time and frequency domains. MSWT offers detailed transient features of a signal and allows for simultaneous analysis in both time and frequency domains. With a scaling parameter a and a translation parameter b, the wavelet transform scales the resolution into capturing high-frequency details at fine scales and low-frequency components at coarser scales. This multi-scale representation proves to be particularly useful in non-stationary signals like EEG, speech, and seismic data wherein features of interest occur at different time spans. MSWT is strong in extracting meaningful features from a signal by noise isolation and pattern identification across scales. It is iterative in handling signal decomposition, producing approximations and details at every level. The approximation captures the wider trends, and the details capture finer, transient features. For example, in biomedical signal processing, MSWT can separate high-frequency noise from low-frequency physiological signals, thus improving the analysis. Its multi-resolution capability is superior to the traditional Fourier-based methods because it preserves time localization.

Equation (1) represents the wavelet function ψ(t) is a small wave like oscillation localized in time and frequency. The wavelet transform applies this function to the signal x(t) by analyzing it at various scales (a) and (b). In this equation a > 0 is a scaling factor, b is a translation factor and $\frac{1}{\sqrt{a}}$ ensures the energy normalization of the scaled wavelet.

1 $${\varphi}_{a,b}\left(t\right)=\frac{1}{\sqrt{a}}\varphi \left(\frac{t-b}{a}\right)$$

The CWT in Eq. (2) is the result of convolution between the signal x(t) and a diluted and shifted form of the wavelet function where ${\varphi \left(a,b\right)}^{*}\left(t\right)$ is the complex conjugate of the scaled and translated wavelet.

2 $${W}_{\varphi }(a,b)={\int}_{-\infty }^{\infty }x\left(t\right){\varphi \left(a,b\right)}^{*}\left(t\right)dt$$

The substitution of the scaled and translated wavelet is given. By factor out the normalization term, the translated wavelet is given in Eq. (3).

3 $${W}_{\varphi }(a,b)=\frac{1}{\sqrt{a}}{\int}_{-\infty }^{\infty }x\left(t\right){\varphi }^{*}\left(\frac{t-a}{a}\right)dt$$

By applying the Eq. (4) and (5), the Eq. (6) has been changed and the it will be simplified by canceling ‘a’ from the denominator and numerator. The final form of the wavelet transforms after the change of variables.

4 $$u=\frac{t-b}{a}$$

5 *t=au+banddt=adu*

6 $${W}_{\varphi }(a,b)=\frac{1}{\sqrt{a}}{\int}_{-\infty }^{\infty }x\left(au+b\right){\varphi }^{*}\left(u\right)du$$

### Kalman filtering with wavelet denoising for noise reduction

Kalman Filtering with Wavelet Denoising combines two powerful techniques to improve signal quality, especially in noisy environments. The Kalman filter is an optimal recursive estimator that predicts the state of a system and corrects it based on noisy observations. It is very effective in scenarios where the signal dynamics are uncertain, and noise is Gaussian in nature. The Kalman filter, when applied in the signal processing environment, offers a method for making incremental, iterated estimates about signal state using a noisy stream of measurements and would suit such time-sensitive applications. Meanwhile, in wavelet denoising, the noise present on different scales of scale representations can be filtered with very less distortion to the desired original signal. It improves the performance by first applying wavelet transform that decomposes the signal into various frequency bands and then applies the Kalman filter for refining estimates and further reduces the noise effect.

Kalman filtering in combination with wavelet denoising enhances the overall signal-to-noise ratio (SNR) and preserves all important features of the original signal. Wavelet denoising identifies and suppresses the noise components that are located at specific scales or frequencies. Kalman filtering uses dynamic state estimation in tracking and predicting the signal much better over time. This is particularly useful in applications such as EEG signal processing where high-frequency noise can completely mask the brainwave patterns. Kalman filtering applied after wavelet-based denoising will correct any residual noise that may remain after the initial wavelet Thresholding by giving a more accurate representation of the signal.

In Eq. (7), the wavelet transform is a signal x(t) is represented where *φ* a,b (t) is the wavelet function at scale a and translation b and ${\sum}_{a,b}{{\varphi }^{*}}_{\left(a,b\right)}\left(t\right)$ represents the multiscale decomposition over different scales.

7 $$x\left(t\right)={\sum}_{a,b}{{\varphi }^{*}}_{\left(a,b\right)}\left(t\right)$$

DWT is highly utilized in practical applications. Decompose the signal into low-frequency components, known as approximation, and high-frequency components, known as detail. Noise tends to be concentrated in the high-frequency components, which are then thresholded. Set a threshold τ such that wavelet coefficients smaller than τ are put to zero. The remaining coefficients are changed with respect to the threshold value. The equation (8) represents the Thresholding factor which reduces the small wavelet coefficients.

8 $$\widehat{x}\left(t\right)={\sum}_{a,b}Threshold\left({{\varphi }^{*}}_{\left(a,b\right)}\left(t\right),\pounds \right)$$

The Kalman filter is a two-stage recursive state estimation algorithm, namely Prediction and Update. Given a system’s state at time k, the Kalman filter predicts the state at time k + 1.This is achieved using the system’s state transition model using the Eq. (9) where *A* is the state transition matrix, ${\widehat{x}}_{k}^{-}$ is the current state estimation, *B* is the control input matrix and ${u}_{k}$ is the control input.

9 $${\widehat{x}}_{k+1}^{-}=A{\widehat{x}}_{k}^{-}+B{u}_{k}$$

The prediction of the error covariance is performed using the Eq. (10) where *Q* is the process noise covariance.

10 $${\widehat{P}}_{k+1}^{-}=A{P}_{k}{A}^{T}+Q$$

Once a new measurement ${z}_{k+1}$ is available , the kalman filter updates the predicted state and error covariance where ${K}_{k+1}$ is kalman gain, H is the measurement matrix and R is the measurement will have in the update.

11 $${K}_{k+1}=\frac{{\widehat{P}}_{k+1}^{-}{H}^{T}}{H{\widehat{P}}_{k+1}^{-}{H}^{T}+R}$$

The state estimate is updated using the equation (12) and the error covariance is updated using the equation (13) where I is the identify matrix.

12 $${\widehat{x}}_{k+1}^{-}={\widehat{x}}_{k+1}^{-}+{K}_{k+1}({z}_{k+1}-H{\widehat{x}}_{k+1}^{-})$$

13 $${P}_{k+1}=\left(I-{K}_{k+1}H\right){P}_{k+1}^{-}$$

### Spectral entropy analysis for epoch detection

Spectral entropy is the method to estimate uncertainty or disorder in a frequency domain of a signal. It is derived from the concept of Shannon entropy that calculates the amount of information or uncertainty contained within a system. In the epoch detection in time-series data such as EEG signals, the usage of spectral entropy allows segmentation into meaningful epochs based on the change in the signal’s frequency content. This procedure initially transforms the signal from its time domain to frequency by methods like Fourier Transformation and Wavelet Transformation and thereafter the signal can represent spectral components. Spectral entropy is then calculated through using the equation (14).

14 $$H\left(f\right)=-{\sum}_{i=1}^{N}p\left({f}_{i}\right)logp\left({f}_{i}\right)$$

where *p*(*f*_*i*_) is the normalized power spectrum at frequency *f*_*i*_ and N is the number of frequency bins. The entropy value (*f*) reflects how spread out the power is across the frequency spectrum. A higher entropy value indicates a more complex and variable signal, while a lower entropy value suggests a more predictable and stable signal. Through entropy values, variations over time can be noticed, leading to transitions that are thought to represent epochs representing different phases of activity of the signal. Therefore, spectral entropy turns out to be a significant feature for epoch segmentation purposes.

**Fig. 1:**
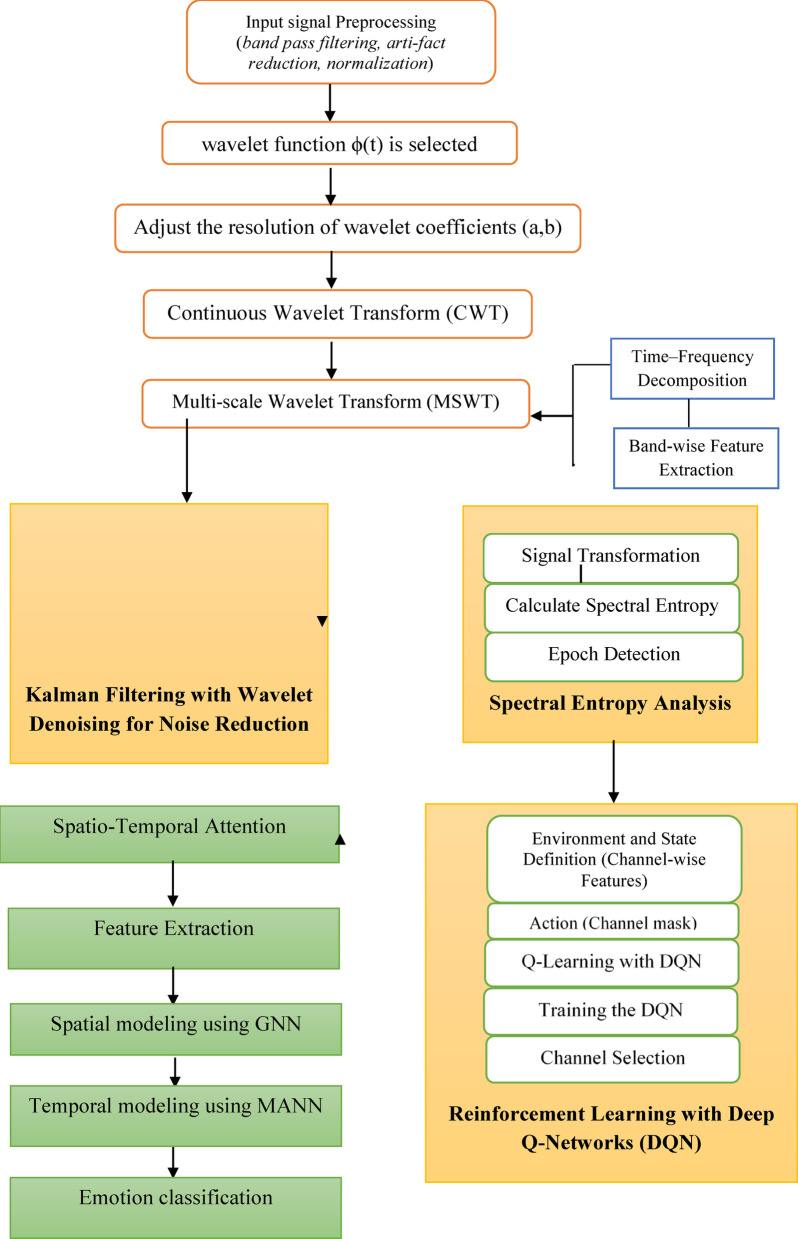
Working flow of the proposed feature extraction.

Figure 1 represents the working flow of the proposed feature extraction process, which extracts relevant features from the input data for further analysis. To start with, the pre-processing step removes noise in the signal and normalizes the data. MSWT decomposes the signal into multiple bands of frequency to capture all the details at different scales. It then applies wavelet denoising to reduce the high-frequency noise in the signal, making it more clear. At the same time, a Kalman filter is used to refine the signal by state estimation to correct residual noise.

Spectral entropy analysis is used to find meaningful epochs or transitions in the signal by analyzing its frequency content. Finally, reinforcement learning with Deep Q-Networks (DQN) is implemented for dynamic decision-making purposes as well as spatio-temporal attention networks used in feature extraction, focusing attention to the most important temporal and spatial features to enhance the accuracy of downstream classification or detection tasks.

### Reinforcement learning with deep Q-networks (DQN) for adaptive channel selection

Reinforcement learning (RL) is a sub-discipline of machine learning in which an agent makes decisions based on its interactions with the environment and as a result is conditioned (reinforced), as well as provided feedback (reward). When applied to adaptive channel selection, RL agent is able to adapt the channel selection strategy as a dynamic way to optimum performance measure, e.g. signal-to-noise ratio (SNR) or throughput.The channel selection process is articulated as Markov Decision Process (MDP) and improved using a Deep Q-Network (DQN). The state demonstration includes channel-wise spectral entropy, band energy, and temporal variance features removed from multi-scale wavelet-decomposed EEG signals. Actions indicate the process of producing a binary channel selection mask and reward function is well-defined based on developments in validation accuracy with a sparsity constraint to dampen the process of redundant channel selection. Through this origination, channel selection is incorporated with feature extraction and classification in an endwise manner.

The goal of the agent is to find the best policy of selecting channels based on the condition of the environment, which can be the avenues of channels and prevailing interference. Another common RL algorithm used in this task is the Deep Q-Network (DQN) that integrates deep learning and Q-learning. In Q-learning, the agent learns a Q-value function that estimates the expected cumulative reward for a given state-action pair. The Q-value is updated iteratively using the Bellman Eq. (17) where ${r}_{t}$ is the immediate reward after taking action ${a}_{t}$. γ is the discount factor that controls the importance of future rewards and α is the learning rate that controls how much new information dominates old information.

17 $$Q\left({s}_{t},{a}_{t}\right)=Q\left({s}_{t},{a}_{t}\right)+\alpha ({r}_{t}+\gamma Q\left({s}_{t+1},a{\prime})-Q\left({s}_{t},{a}_{t}\right)\right)$$

In DQN, the neural network is used to approximate the Q-value function; hence, it is possible for the model to manage a high-dimensional and complex state space. The Q-network is trained using the following loss function in Eq. (18) where *θ* represents the parameters of the network and ${\theta }^{-}$ represents the parameters of a target network that is periodically updated to stabilize training. The agents policy is derived from the Q values by selecting the action ${a}_{t}$ that maximizes the Q value.

18 $$L\left(\theta \right)=E\left[{({r}_{t}+\gamma Q\left({s}_{t+1},{a}{\prime};{\theta }^{-})-Q\left({s}_{t},{a}_{t};\theta \right)\right)}^{2}\right]$$

19 $${a}_{t}=argargQ({s}_{t},{a}_{t};\theta )$$

Equation (19) corresponds to selecting the channel that maximizes the expected reward. Using DQN for adaptive channel selection, the agent learns to adapt to the variability in environmental conditions such as the fluctuation of channel availability or interference levels. Over time, the model becomes proficient in selecting channels that maximize long-term rewards, such as throughput or SNR, while minimizing interference. This dynamic adaptation allows the agent to navigate efficiently through changing network conditions, which makes it very suitable for real-time systems in wireless communication or signal processing. Moreover, DQN is able to deal with multi-dimensional input states such as network congestion, interference patterns, and channel characteristics, so it is suitable for complex and highly dynamic environments.

### Spatio-Temporal attention networks (ST-ANs) for feature extraction

Spatio-Temporal Attention Networks (ST-ANs) are aimed at capturing spatial and temporal dependencies in sequential data in tasks such as video, time-series, or multi-dimensional signals like EEG. This is based on the fact that the core idea of ST-ANs lies in integrating attention mechanisms such that the network can concentrate on the most informative parts in both the spatial (like different regions of a signal) and temporal (such as key time steps) dimensions. Through attention layers, the model is learned to pay different attentions to spatial locations and time frames. This approach will highlight the critical features while suppressing the irrelevant information and making the ST-ANs highly efficient in extracting features from dynamic complex data environments.

Equation (20) focus on the most relevant time steps across the entire sequence. The temporal attention weight is computed by applying a similar mechanism but across all time stpes.in this, W is the weight ,b is a bias and x is an input value.

20 $${\alpha}_{t}^{spatial}=softmax\left({W}_{spatial}{x}_{t}+{b}_{spatial}\right)$$

Equation (21) focus on the most relevant time steps across the entire sequence. The temporal attention weight is computed by applying a similar mechanism but across all time steps.

21 $${\alpha}_{t}^{temporal}=softmax\left({W}_{temporal}{x}_{t}+{b}_{temporal}\right)$$

The final spatio temporal attention weight in Eq. (22) combines both spatial and temporal attention. This results in a weighted feature representation that emphasizes both important spatial and temporal aspects of the input sequence.

22 $${\alpha}_{t}^{ST}={\alpha}_{t}^{spatial}+{\alpha}_{t}^{temporal}$$

After computing the attention weights, the final output of the ST-AN’s is obtained by weighting the feature vector at each time step with the spaito temporal attention as given in Eq. (23). The aggregated sequence of features can then be passes to the subsequent layers of the mode for classification.

23 $${\widehat{x}}_{t}={\alpha}_{t}^{ST}.{x}_{t}$$

### Ensemble of graph neural networks (GNNs) and memory-augmented neural networks (MANNs) for emotion classification

The Ensemble of Graph Neural Networks (GNNs) and Memory-Augmented Neural Networks (MANNs) approach combines the strengths of both GNNs and MANNs to improve emotion classification tasks. GNNs are very good at capturing structural relationships between different elements in data, which is done by representing them as graphs where nodes are data points (such as EEG signals or images) and edges represent relationships or interactions between them. This ensemble approach uses GNNs to model dependencies between different features or nodes by letting the network to learn spatial and relational patterns from raw data. It is very useful for emotion recognition because emotional cues might be scattered around various regions of the input data and understanding the relation between them. The GNN-based model processes the graph structure and propagates information across nodes to obtain enriched feature representations.

Then comes the Memory-Augmented Neural Networks, which enhance the capabilities of long-term storage and retrieval so are more useful in storing memory of sequences or events along periods in a task. For emotions classifications, MANNs well capture long dependencies in times-series data such as those represented by EEG signals when states of emotion evolve through time. By incorporating an external memory component, MANN improves its model’s ability to recall previous information relevant to the current state while also using it appropriately when making accurate classifications.

The selection of Graph Neural Networks (GNNs) and Memory-Augmented Neural Networks (MANNs) is motivated by the intrinsic spatial–temporal nature of EEG signals. EEG electrodes show non-Euclidean spatial relationships, where emotional evidences arise from connections between different regions of brain rather than inaccessible channels; GNNs effectually capture the dependencies deprived of imposing artificial sequential ordering, contrasting to RNNs or Transformers. MANNs capture long-term temporal dependencies and subject-specific emotional patterns with the help of an external memory mechanism, thus trying to extract noticeable historical evidence beyond static hidden states. Together, the amalgamation of GNNs and MANNs empowers joint learning of spatial connectivity and persistent temporal context, aligning well with neurophysiological features of emotion-related EEG dynamics.

Table 2 outlines the architecture of the proposed ensemble model that combines GNN with MANN for emotion classification. The model begins with an input layer that feeds graph data, with the shape (None, 2548, features), where each node has a specific set of features.

**Table 2:** A model summary of the proposed work.

Layer	Output Shape	Param #
Input layer	(None, 2548, features)	0
Lambda (GNN)	(None, 2548, features, 1)	0
Graph Convolution (GNN)	(None, 2548, hidden_dim)	Hidden_dim * features + hidden_dim * 2548
Activation (GNN)	(None, 2548, hidden_dim)	–
Graph Convolution (GNN)	(None, 2548, hidden_dim)	Hidden_dim * hidden_dim + hidden_dim * 2548
Activation (GNN)	(None, 2548, hidden_dim)	–
Pooling (Optional, GNN)	(None, pooled_nodes, hidden_dim)	–
Flatten (GNN)	(None, flattened_dim)	0
GRU (MANN)	(None, flattened_dim, 256)	198,912
Flatten (MANN)	(None, flattened_dim * 256)	0
Dense (MANN)	(None, 3)	1,956,867

The GNN component starts with a Lambda layer which reshapes the input data, followed by two graph convolution layers that capture spatial dependencies across the nodes. After each convolution, an activation function is applied to introduce non-linearity. Optionally, a pooling layer reduces the size of the graph representation and it is flattened into a vector of size 1D. The flattened representation of the node-wise feature vectors is fed into the MANN part of the model, which consists of a GRU layer to capture the temporal dependencies followed by a flattening again, and finally applies the dense layer for the classification in three emotion classes. This architecture takes the advantage of both GNNs at graph-based data and MANNs for sequential feature processing.

**Fig. 2:**
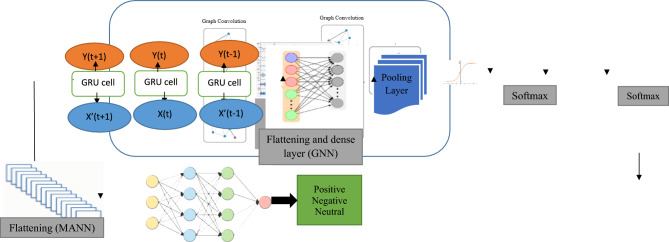
An architecture of ensemble graph neural networks (GNNs) and memory-augmented neural networks (MANNs).

Figure 2 illustrates the architecture of ensemble model that combines GNN and MANN for emotion classification. The model is divided into an input layer that processes graph data with node features. The GNN architecture applies graph convolution layers so that spatial dependencies can be captured within the graph’s structure, followed by activation functions to introduce non-linearity.

The output from the GNN is then flattened and passed into MANN, which applies a Gated Recurrent Unit layer for the modeling of sequential dependencies while further refining the features. The model will then output a dense classification layer to map the input to one of three classes. The ensemble approach takes the strengths of both GNNs for graph-based learning and MANNs for sequential data processing, making it suitable for tasks like emotion classification from graph-structured data. Reinforcement learning based channel selection is implemented in order to improve the performance of emotional state classification as well as to reduce the impact of subject –dependent noise and extraneous channels. These adaptive strategies are helpful on extracting the most relevant patterns across subjects instead of the model dependency on static electrode configuration. Moreover, the GNN module seizures inter-channel relationships which persist moderately stable across individuals, whereas the MANN component conserves long-term temporal context connected with emotional states. Altogether, this integration endorses feature representations that are less delicate to individual differences, causing an improved subject-independent performance not required of domain adaptation or meta-learning strategies.

## Results and discussion

### Dataset 1: EEG brainwave dataset: feeling emotions

This EEG Brainwave dataset captures positive, neutral, and negative emotional experiences by way of brain signals acquired with the Muse EEG headband. The headband has a recording capability of four placements for electrodes, namely TP9, AF7, AF8, and TP10. This recording is made with dry electrodes that are less invasive in nature. Data were gathered from two subjects: a male and a female, both for three minutes in every emotional state^12^.

### Dataset 2: DEAP dataset

DEAP dataset is a large collection meant for studies in affective computing with physiological and EEG recordings. The dataset is split into two parts: ratings from the online self-assessment of 120 extracts from music videos and an experiment with 32 participants watching a subset of 40 videos. Ratings on arousal, valence, and dominance were provided by the participants, making the dataset multidimensional^12^. In Table 3, Valence is the primary measure of emotional polarity, where high valence (≥ 5) indicates pleasant emotions (joy), and low valence (< 5) represents unpleasant emotions (sadness). Arousal distinguishes between active and passive states where high arousal (≥ 5) reflects energetic emotions like anger and low arousal (< 5) corresponds to passive states such as calmness. Dominance is the degree of control felt in an emotion, and high dominance (≥ 5) means empowering emotions such as confidence while low dominance (< 5) refers to submissive feelings such as fear. Together, these dimensions provide a multidimensional framework to classify emotions.

**Table 3:** Emotion classification from valence, arousal, and dominance.

Emotion category	Valence	Arousal	Dominance
Positive	High (≥ 5)	Moderate to High (≥ 5)	High (≥ 5)
Negative	Low (< 5)	Moderate to High (≥ 5)	Low (< 5)
Neutral	Mid-range (~ 5)	Low to Moderate (< 5)	Moderate (around 5)

### Dataset 3: EDA on emotion recognition using EEG (S01G1AllChannels)

This dataset contains time series recordings from all channels of EEG for one participant-S01G1. The data were arranged in a CSV structure where each row was designated to one channel and different columns corresponded to time point in the trial. It is especially well-suited for channel selection and feature extraction experiments, because its single-subject focus allows the exploration of temporal dynamics in EEG signals in greater detail^12^. The detailed dataset description is given in Table 4.

**Table 4:** Dataset description.

Dataset	Total data count	Training data count	Testing data count	Validation data count
EEG Brainwave Dataset	540 data points	324 (60%)	108 (20%)	108 (20%)
DEAP Dataset	1,280 trials	768 (60%)	256 (20%)	256 (20%)
S01G1AllChannels.csv	128 data points	77 (60%)	26 (20%)	25 (20%)

### Performance analysis of proposed work on EEG brainwave dataset

**Fig. 3:**
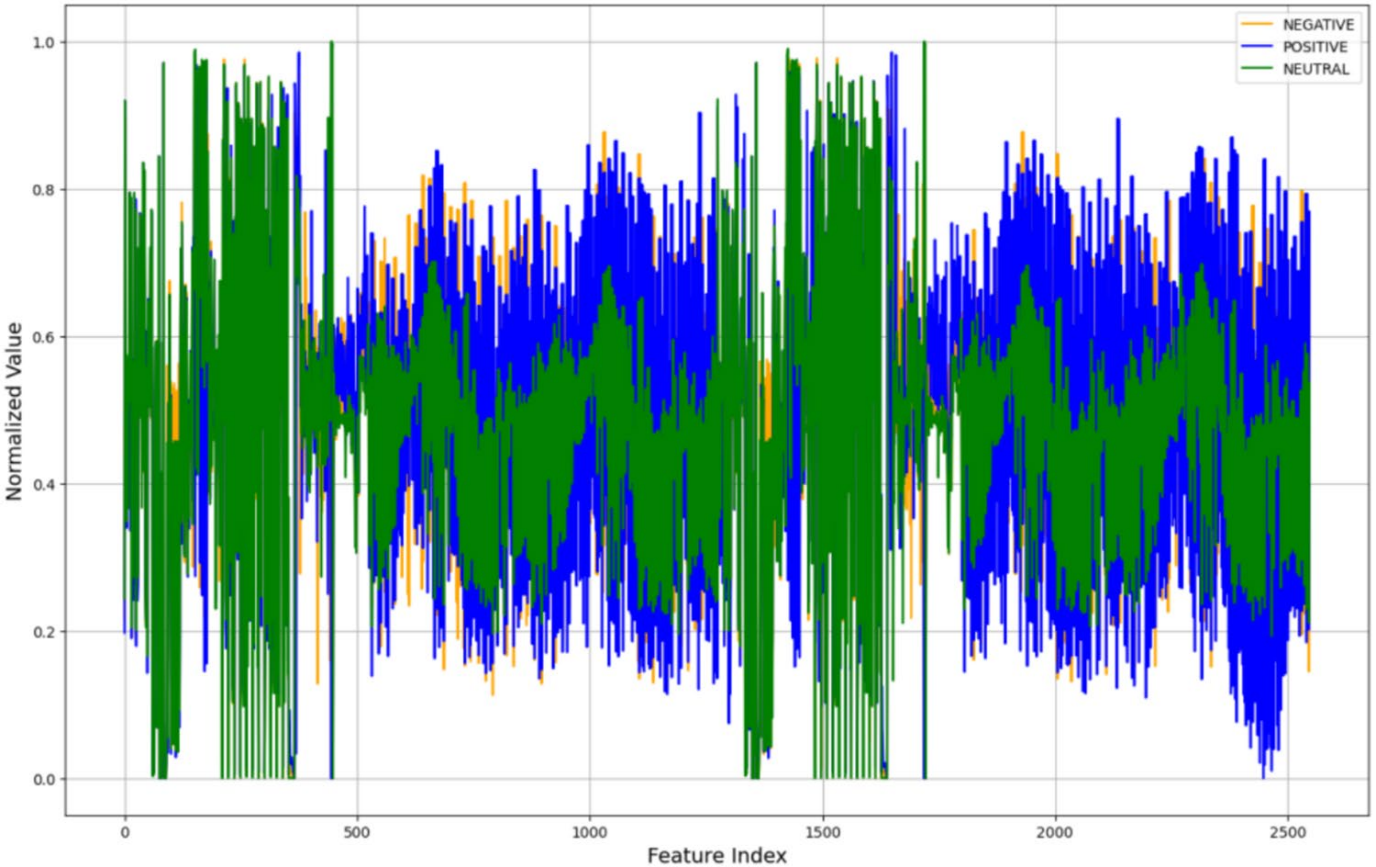
Sample EEG signal for each emotion.

**Fig. 4:**
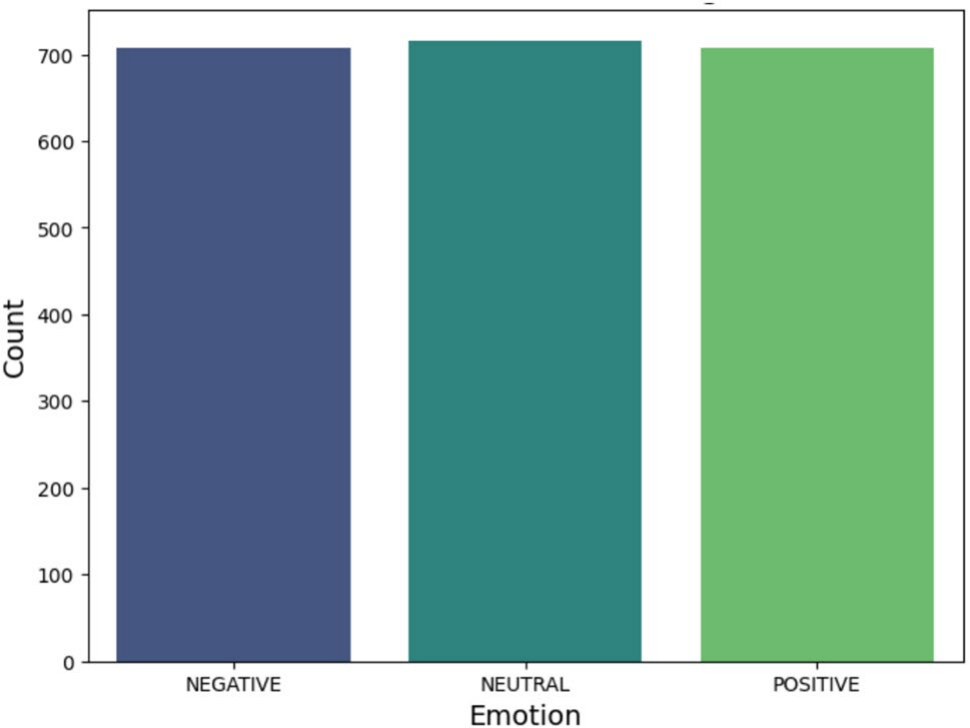
Distribution of emotion categories.

**Fig. 5:**
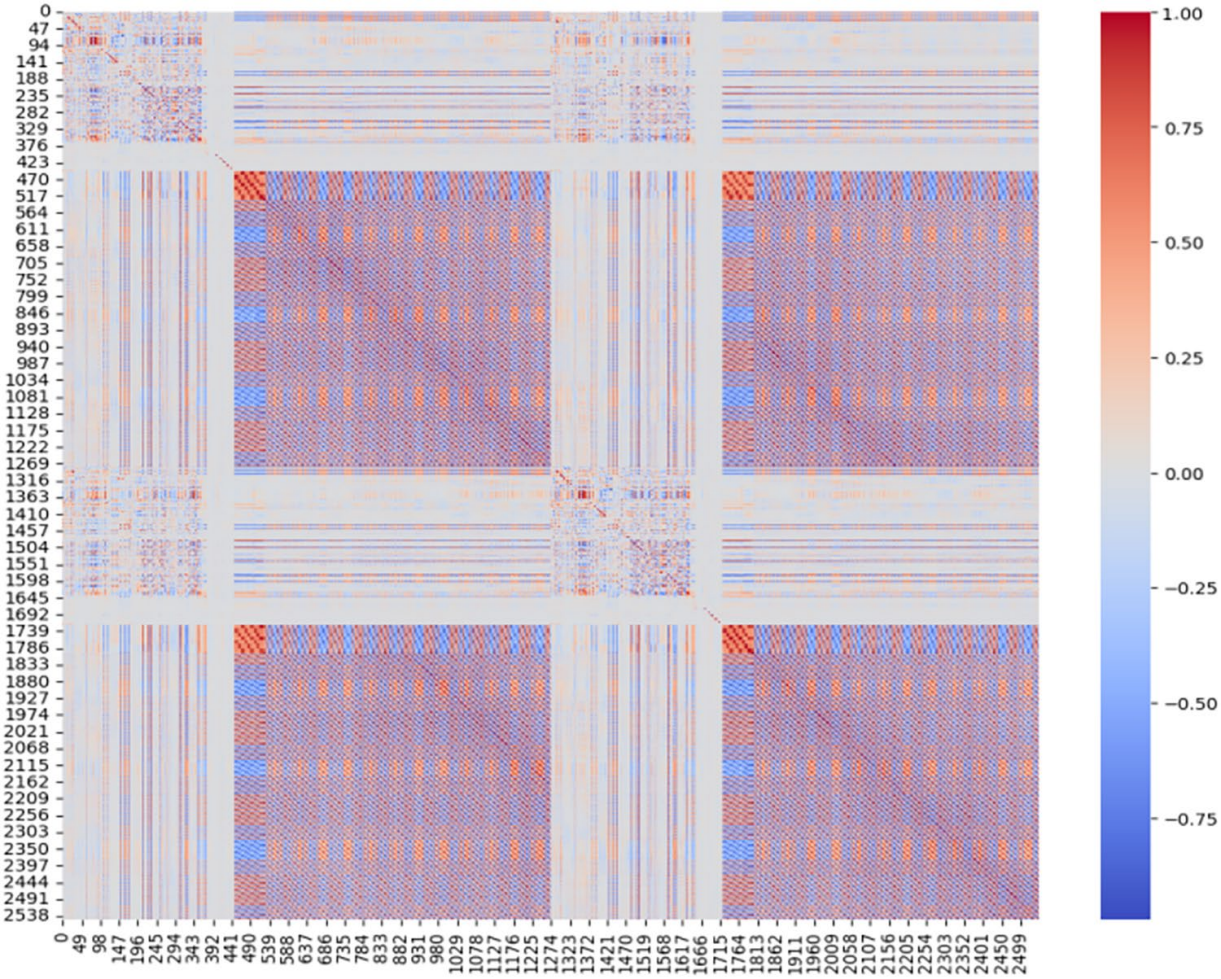
Feature correlation heat map.

**Fig. 6:**
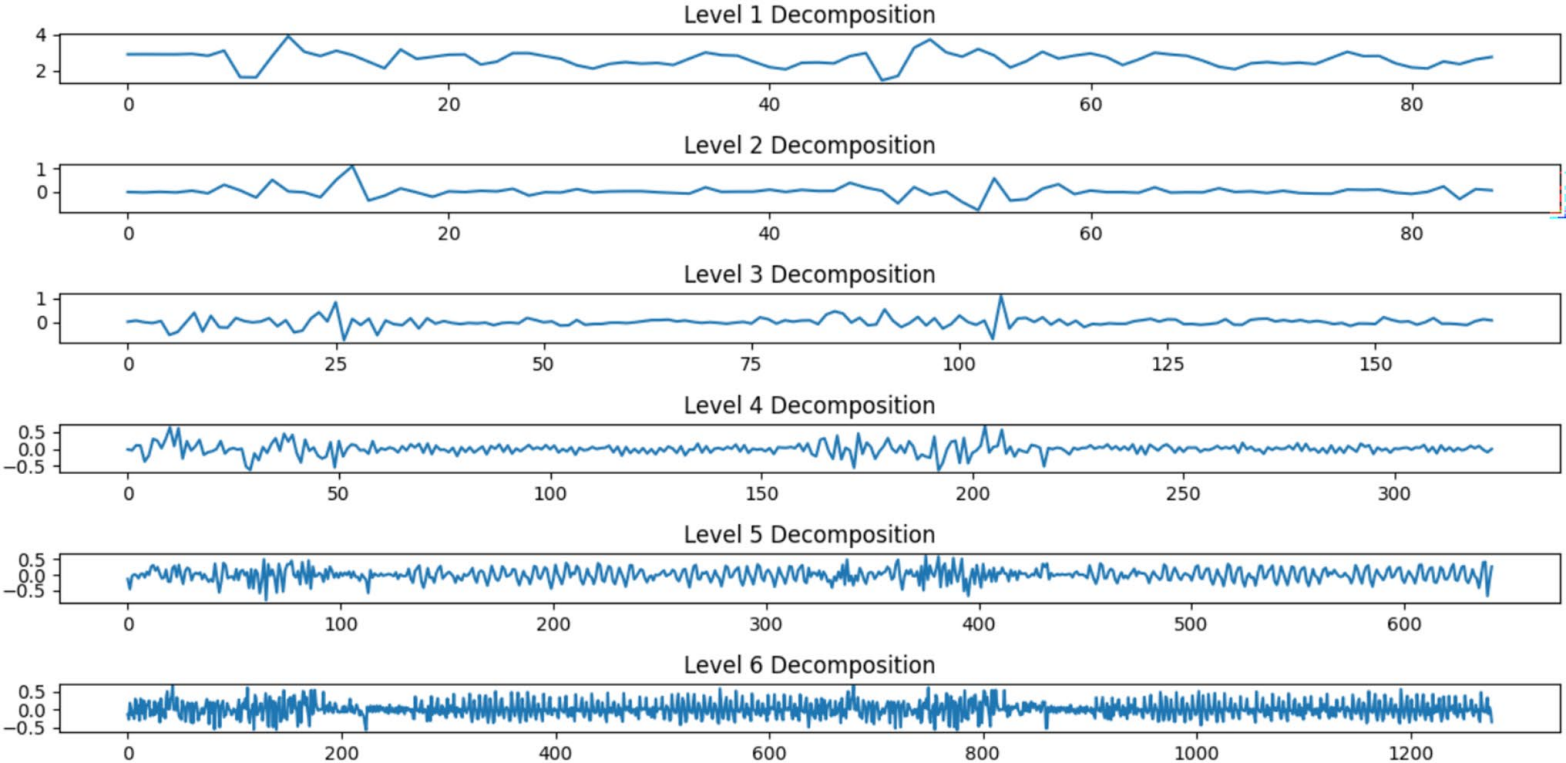
Representation of multi-scale wavelet transform (MSWT), applied to an EEG signal, implies the decomposition of high –frequency to long-duration components.

Figure 3 shows raw EEG signals for each emotion and shows the patterns that occur with each emotion. It gives an idea of how the brain activities are different from one emotional response to another. Figure 4 illustrates the distribution of various categories within the dataset, showing a percentage that has occurred for each emotional state. This gives a scope on the balance or imbalance of the emotion classes in analysis. Figure 5 displays a heat map depicting the correlation between various features from the EEG signals. Such heatmaps are helpful to find the relationship between all components of the signal; otherwise, they can affect classification performance. Figure 6 shows that MSWT is applied in decomposing the EEG signal into multiple scales, where x-axis represents the progression of EEG signal across different times and y-axis denotes the scale of signal components identified at the decomposition level, replicating signal energy at diverse frequency scales. MSWT is effective in performing a time-frequency analysis, capturing high-frequency and low-frequency components of the signal. In MSWT, level 1 denotes the maximum frequency, minimal signal strength with the optimum details. As the level expands from 2 – 6, signal develops more down sampled version representing lower-frequency components.This ordered decomposition expedites multi-scale investigation of EEG dynamics.

**Fig. 7:**
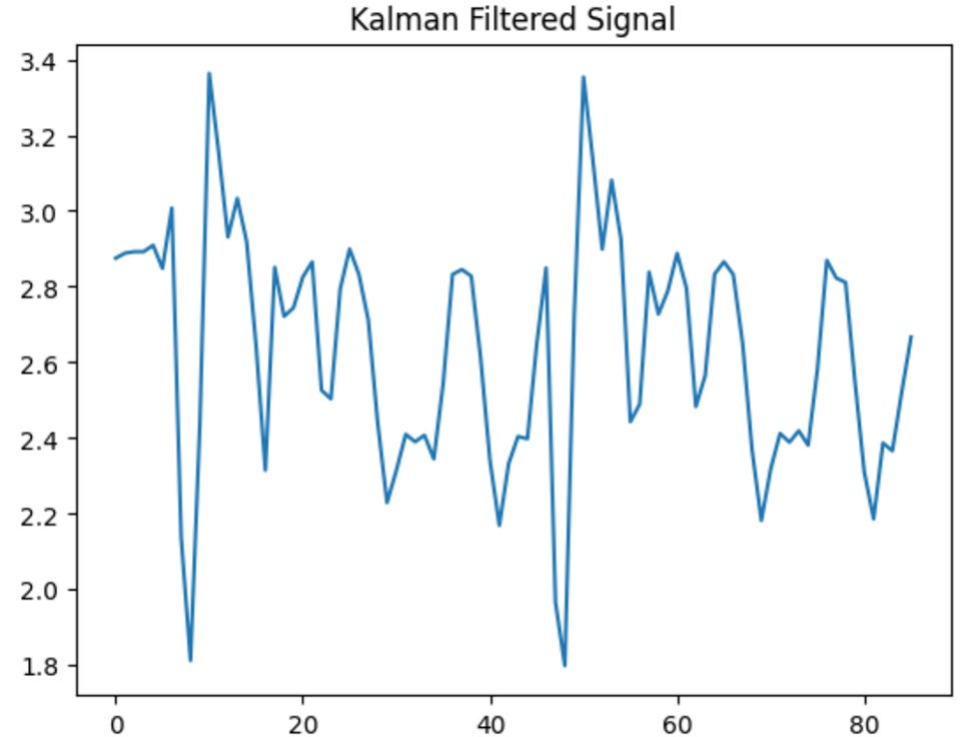
Integrated Kalman filtering and wavelet denoising framework for noise suppression and improving the quality of EEG signal.

**Fig. 8:**
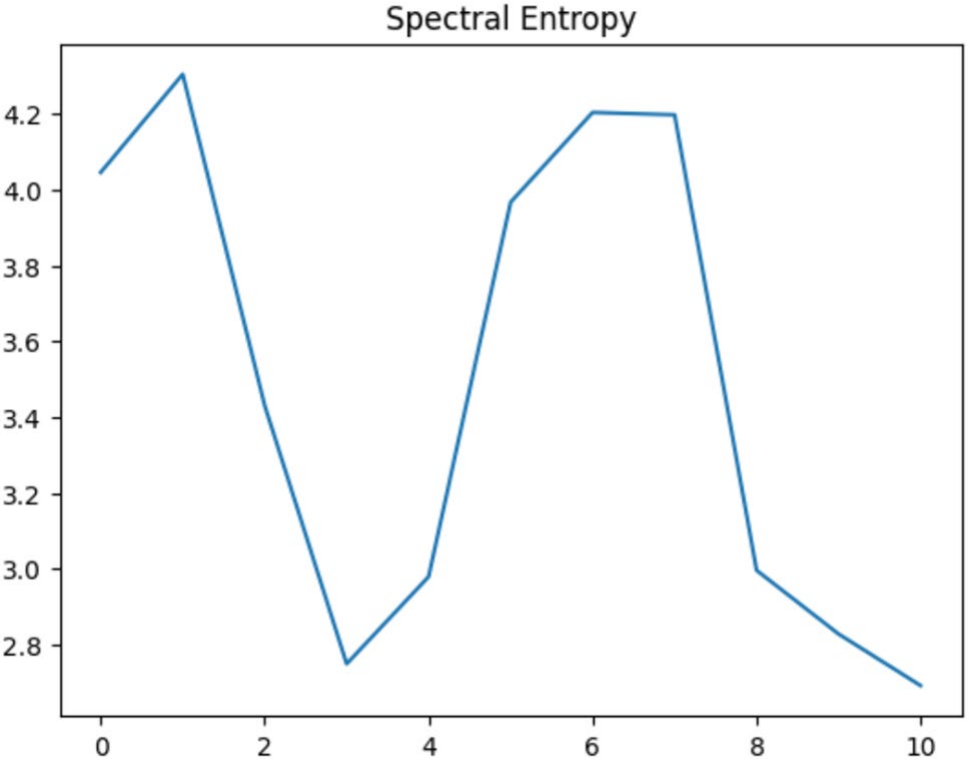
Spectral entropy analysis for automatic detection and segmentation of signal epochs with respect to frequency distribution.

Figure 7 displays how the Kalman filtering would combine with wavelet denoising to enhance signal quality, across different time samples (x-axis) and signal amplitude (y-axis) before and after denoising. It reduces the noise effectively and enhances clarity for the EEG signals, critical to detecting emotions accurately. Figure 8 helps to detect specific epochs (x-axis) in the EEG signal by quantifying the signal’s complexity, representing the low/high spectral entropy value (y-axis). It is important for identifying periods of high emotional shifts in the data.

Table 5 outlines the details of simulation parameters, such as EEG sampling rate, the number of channels, dataset size, emotion categories, and signal decomposition technique. Tables 6 and 7 report the training, validation, and testing results for accuracy, loss, precision, recall, and F1 score, representing the model with excellent performances in high accuracy (96.5%), low loss (0.06), and consistent precision, recall, and F1 scores across categories.

**Table 5:** Experimental configuration and EEG signal acquisition parameters for the EEG brainwave dataset.

Parameter	Description	Value/Setting
EEG Sampling Rate	Frequency of EEG signal sampling	256 Hz
Number of Channels	EEG channels used	TP9, AF7, AF8, TP10
Recording duration	Time taken to record the EEG data	3 min per state per participant
Emotion Categories	Classes -3	Positive, Neutral, Negative
Signal Decomposition Method	Technique for decomposing signals	Multi-Scale Wavelet Transform

**Table 6:** Classification Performance metrics of the proposed model on EEG brainwave dataset across training, validation and testing.

Emotion category	Accuracy (%) (Training / Validation / Testing)	Precision (%) (Training / Validation / Testing)	Recall (%) (Training / Validation / Testing)	F1-score (%) (Training / Validation / Testing)
Positive	99.2 / 95.8 / 94.5	98.7 / 94.5 / 93.3	98.7 / 94.5 / 93.3	98.6 / 94.4 / 93.1
Neutral	98.5 / 96.1 / 92.8	98.0 / 95.0 / 91.2	98.0 / 95.0 / 91.2	98.1 / 95.3 / 91.5
Negative	99.0 / 96.4 / 93.2	98.2 / 95.8 / 92.5	98.2 / 95.8 / 92.5	98.4 / 95.5 / 92.4
Overall	98.5 / 93.1 / 96.5	97.3 / 94.1 / 91.3	98.5 / 95.0 / 92.4	98.4/ 95.1 / 92.3

**Table 7:** Training/ validation / testing loss of the proposed model on EEG brainwave dataset across emotion categories.

Emotion category	Training loss	Validation loss	Testing loss
Positive	0.02	0.05	0.07
Neutral	0.03	0.04	0.06
Negative	0.02	0.05	0.06
Overall	0.02	0.05	0.06

Table 8 compares the proposed model with existing methods, showing a significant improvement in accuracy (98.5%) and loss (0.02) over traditional approaches for dataset 1 and accuracy (94.5%) and loss (0.55%) for dataset 2, highlighting the effectiveness of the ensemble of GNNs and MANNs used in the proposed work. In addition to the subject-independent train-test split, k-fold cross-validation was used to guarantee reliable and objective performance evaluation. The EEG dataset was divided into k about equal-sized, non-overlapping folds. One fold was utilized for testing and the remaining k − 1 folds were used for training in each iteration. To ensure that every fold was used as the test set exactly once, this procedure was repeated k times. In order to reduce volatility and increase the dependability of the presented results, the final performance measures were calculated as the average across all folds.The dataset was split into five equal folds, with the remaining folds being utilized for training and each fold serving as the test set once. To lessen evaluation bias and volatility, this procedure was carried out five times and performance measures were averaged across all folds. The dataset was divided into five non-overlapping folds for the fivefold cross-validation technique. One fold (Folds 1 through 5) served as the test set in each iteration, with the other four folds being utilized for training. The final performance metrics were derived by averaging the results across all folds after this process was repeated five times, with each fold acting as the test set once.

**Table 8:** Comparative analysis of existing methods and proposed work.

S.No	Methods	Accuracy (%) dataset 1	Loss dataset 1	Accuracy (%) dataset 2	Loss dataset 2
1	Traditional bandpass filtering	85.2	0.15	82.4	0.76
2	Kalman filtering with wavelet denoising	90.3	0.12	85.7	0.66
3	ICA (Independent Component Analysis)	88.4	0.13	84.2	0.57
4	Fixed channel selection	85.7	0.16	83.1	0.66
5	Reinforcement learning with DQN	91.5	0.10	89.6	0.62
6	Spatio-Temporal attention networks (ST-ANs)	NA	NA	92.3	0.60
7	Ensemble of GNNs and MANNs	98.5	0.02	94.5	0.55

**Fig. 9:**
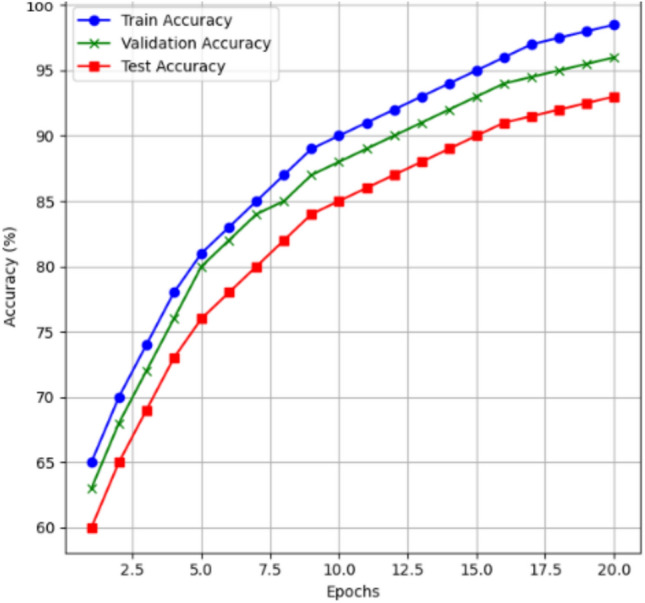
Accuracy curves of proposed ensemble of graph neural networks (GNNs) and memory-augmented neural networks (MANNs).

**Fig. 10:**
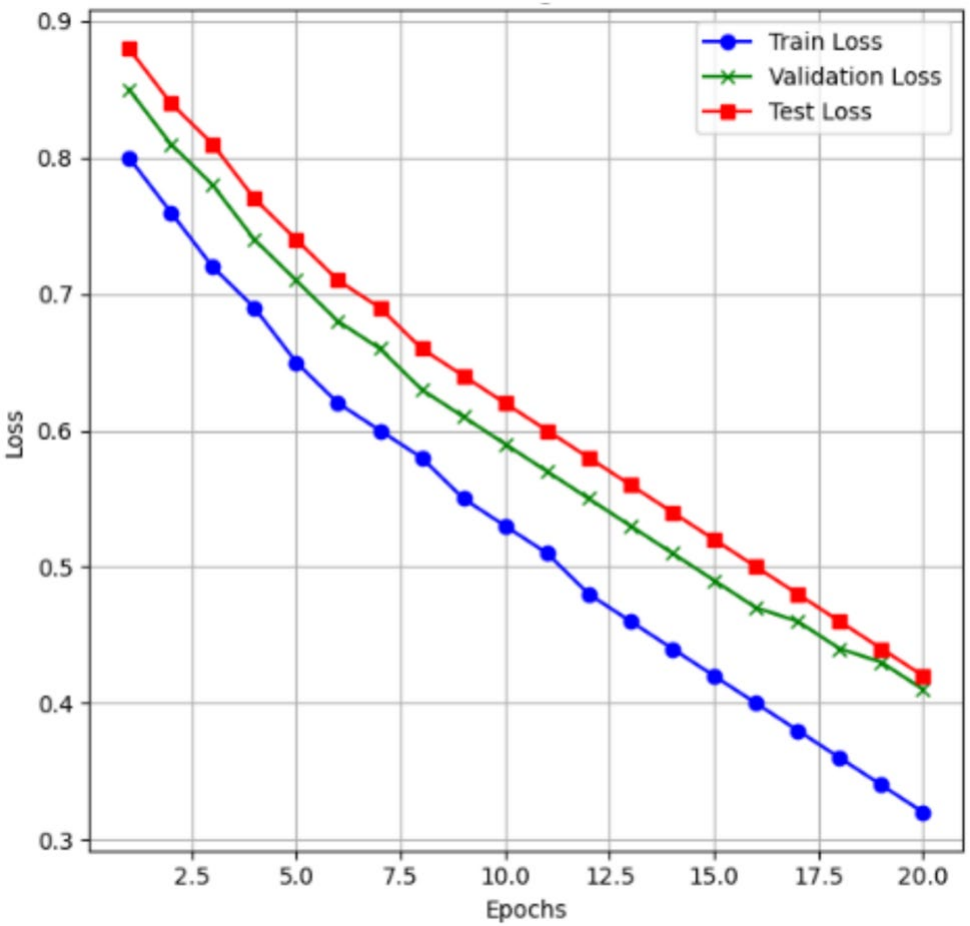
Loss curves of proposed ensemble of graph neural networks (GNNs) and memory-augmented neural networks (MANNs).

Figures 9 and 10 present the performance curves of the proposed Ensemble of Graph Neural Networks (GNNs) and Memory-Augmented Neural Networks (MANNs). Figure 9 plots the accuracy curves for which the model improves its accuracy over training, validation, and testing phases with an upward trend that stabilizes at high values, showing good learning robustness. Figure 10 depicts the drop in loss during training showing how loss gradually decreases to minimal amounts with time.

Instead of overfitting, normalized feature representations and the use of categorical cross-entropy with well-separated class distributions are responsible for the low loss values seen during training (Fig. 9 and 10). Early stopping, dropout, and L2 regularization were used to avoid memorization. Additionally, cross-validation and subject-independent evaluation made sure that performance improvements were due to generalization rather than data leakage.

### Performance analysis of proposed work on EEG DEAP dataset

**Fig. 11:**
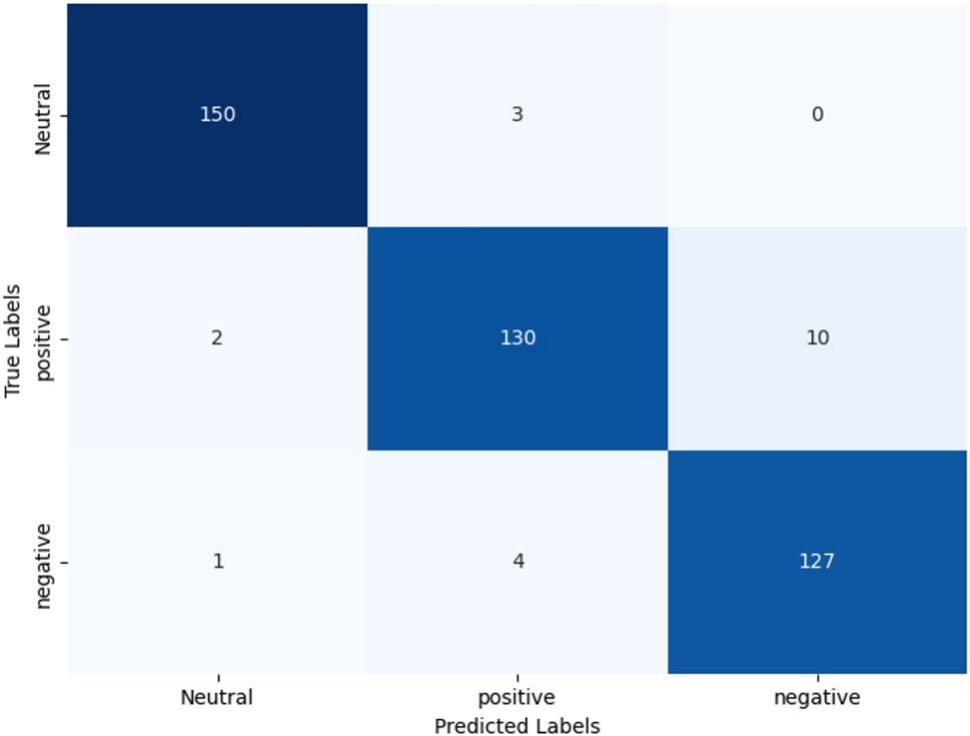
Confusion matrix.

Figure 11 displays the confusion matrix, giving an overall view of how the model classifies its performance by showing the true positive, false positive, true negative, and false negative rates for each category of emotion, thus summarizing the model’s accuracy in predicting the emotional states from EEG signals.

Table 9 compares precision and recall, where the Ensemble of GNNs and MANNs again tops the chart with a precision of 90.3% and recall of 91.1%.

**Table 9:** Precision and recall comparison.

S.No	Methods	Precision (%)	Recall (%)	F1-Score (%)
1	Traditional Bandpass Filtering	79.3	80.1	79.7
2	Kalman Filtering with Wavelet Denoising	82.4	83.7	83.0
3	ICA (Independent Component Analysis)	81.2	82.5	81.8
4	Fixed Channel Selection	80.4	81.9	81.1
5	Reinforcement Learning with DQN	85.6	86.4	86.0
6	Spatio-Temporal Attention Networks (ST-ANs)	88.7	89.2	89.4
7	Ensemble of GNNs and MANNs	90.3	91.1	91.0

Table 10 highlights the subject generalization capability, where the Ensemble of GNNs and MANNs demonstrates superior generalization at 85.1%, outperforming all other methods.

**Table 10:** Subject generalization comparison.

S.No	Methods	Subject generalization (%)
1	Traditional Bandpass Filtering	72.3
2	Kalman Filtering with Wavelet Denoising	74.5
3	ICA (Independent Component Analysis)	75.0
4	Fixed Channel Selection	73.8
5	Reinforcement Learning with DQN	80.2
6	Spatio-Temporal Attention Networks (ST-ANs)	82.7
7	Ensemble of GNNs and MANNs	85.1

**Fig. 12:**
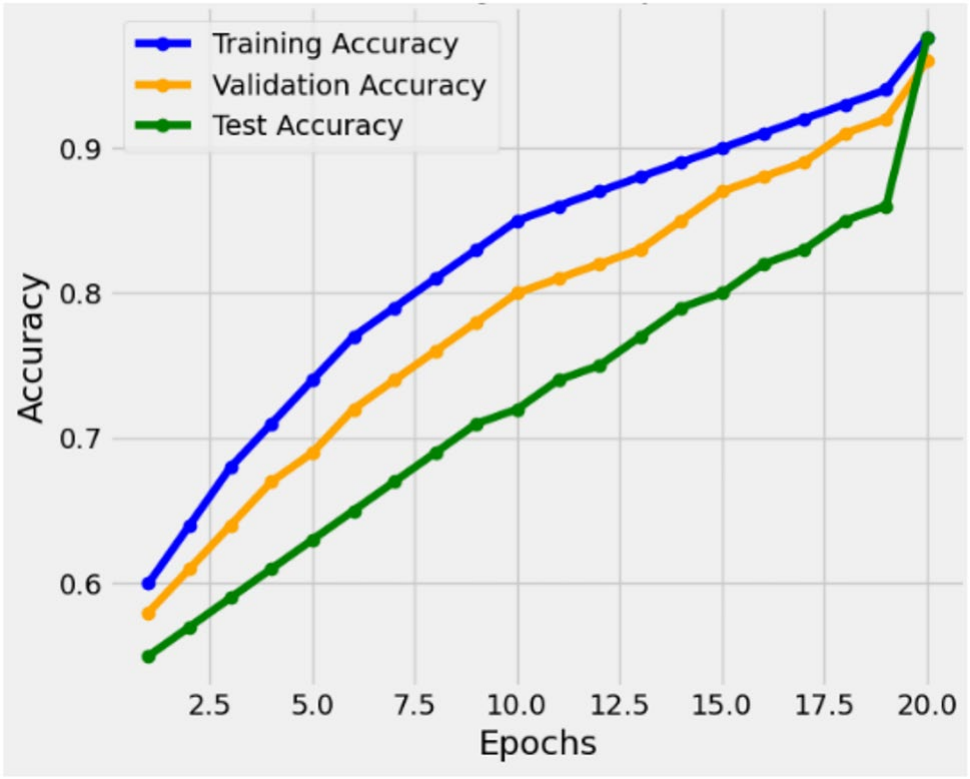
Accuracy of the proposed work.

Figure 12 shows accuracy curve with steady improvement in accuracy of the model during training, validation, and testing phases with effective learning of the model and high precision in the classification task.

**Fig. 13:**
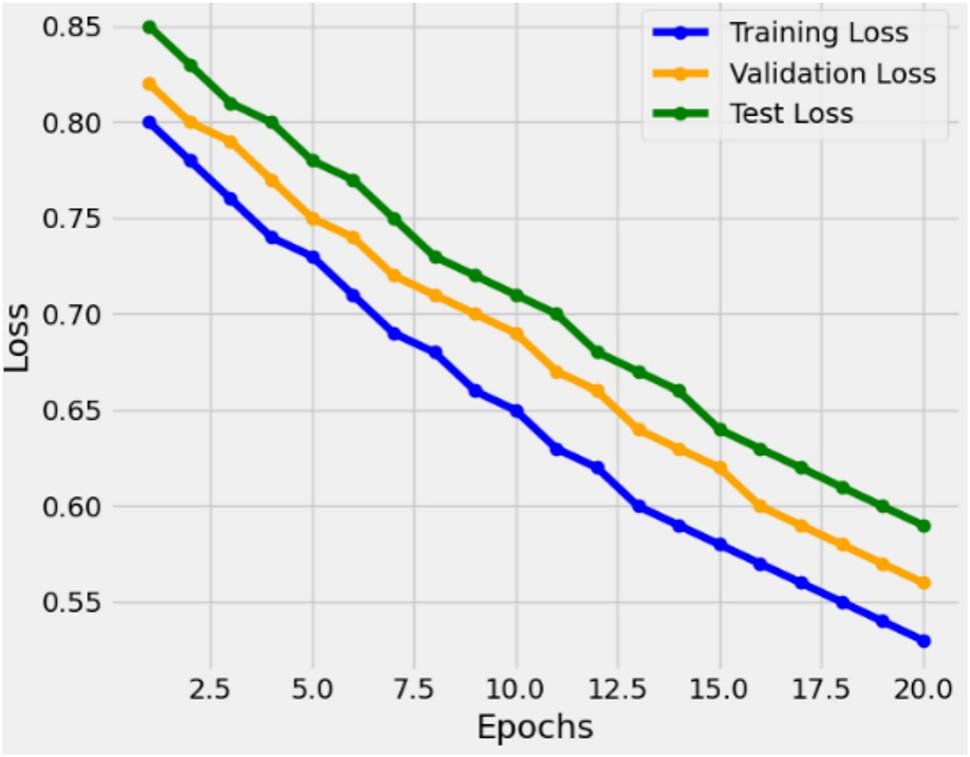
Loss of the proposed work.

Figure 13represents loss curve in which loss goes down steadily and reflects that the optimization is done very efficiently and the model has a very less error during training.

### Performance analysis of proposed work on EEG S01G1allchannels dataset

**Fig. 14:**
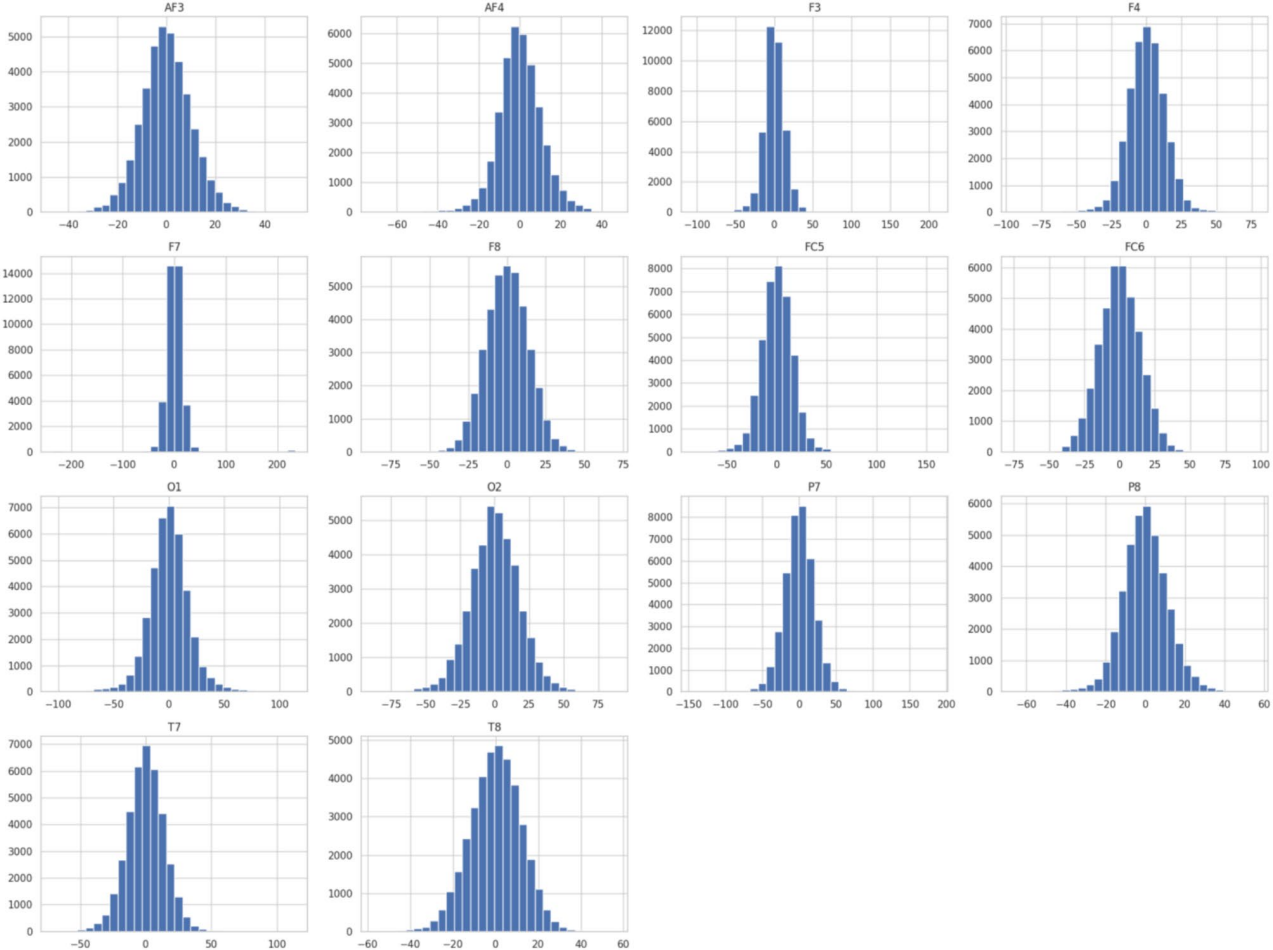
Statistical distribution of extracted EEG features across multiple channels illustrating variability and dispersion characteristics.

**Fig. 15:**
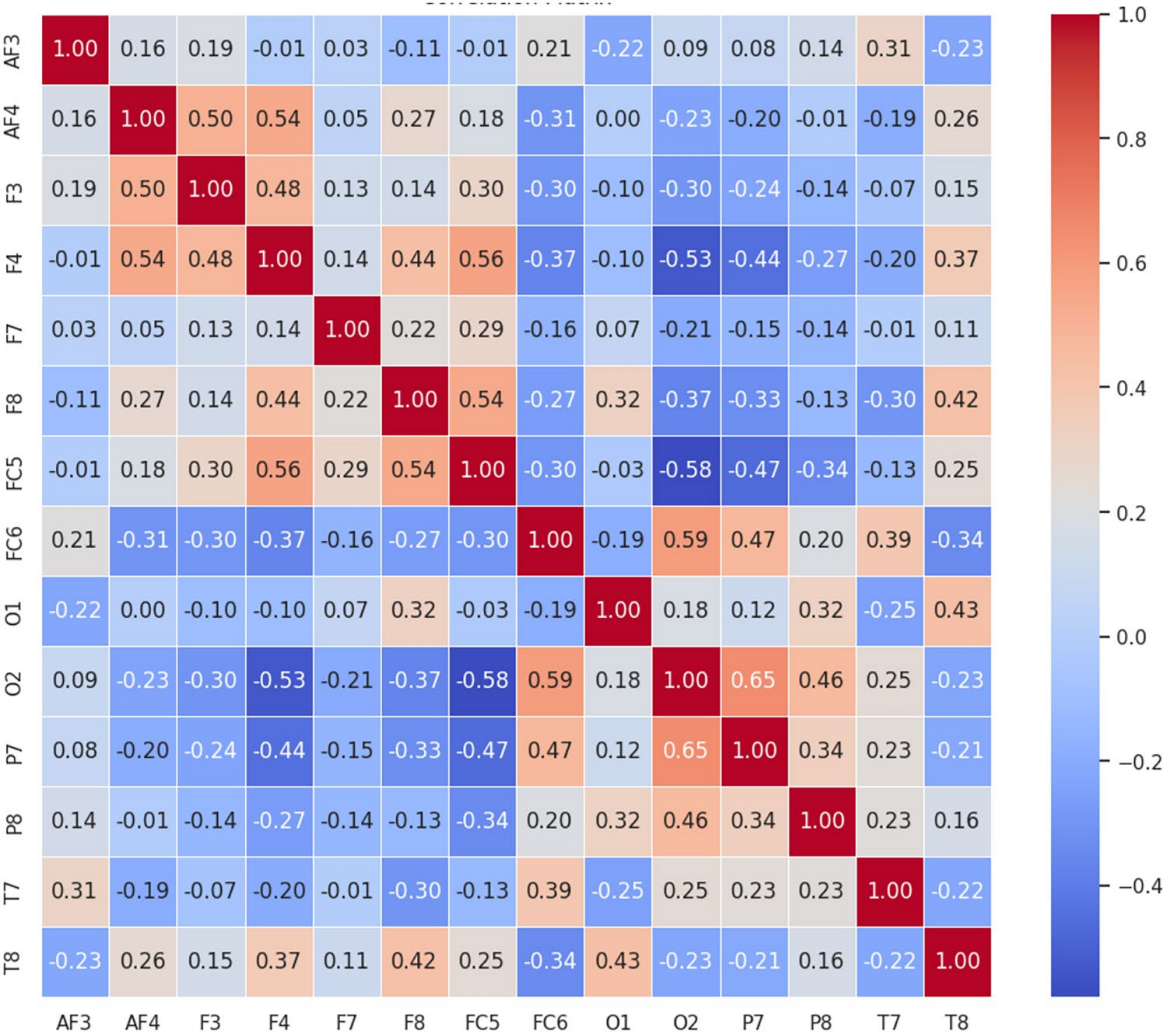
Correlation matrix depicting the strong and weak dependencies among channels.

**Fig. 16:**
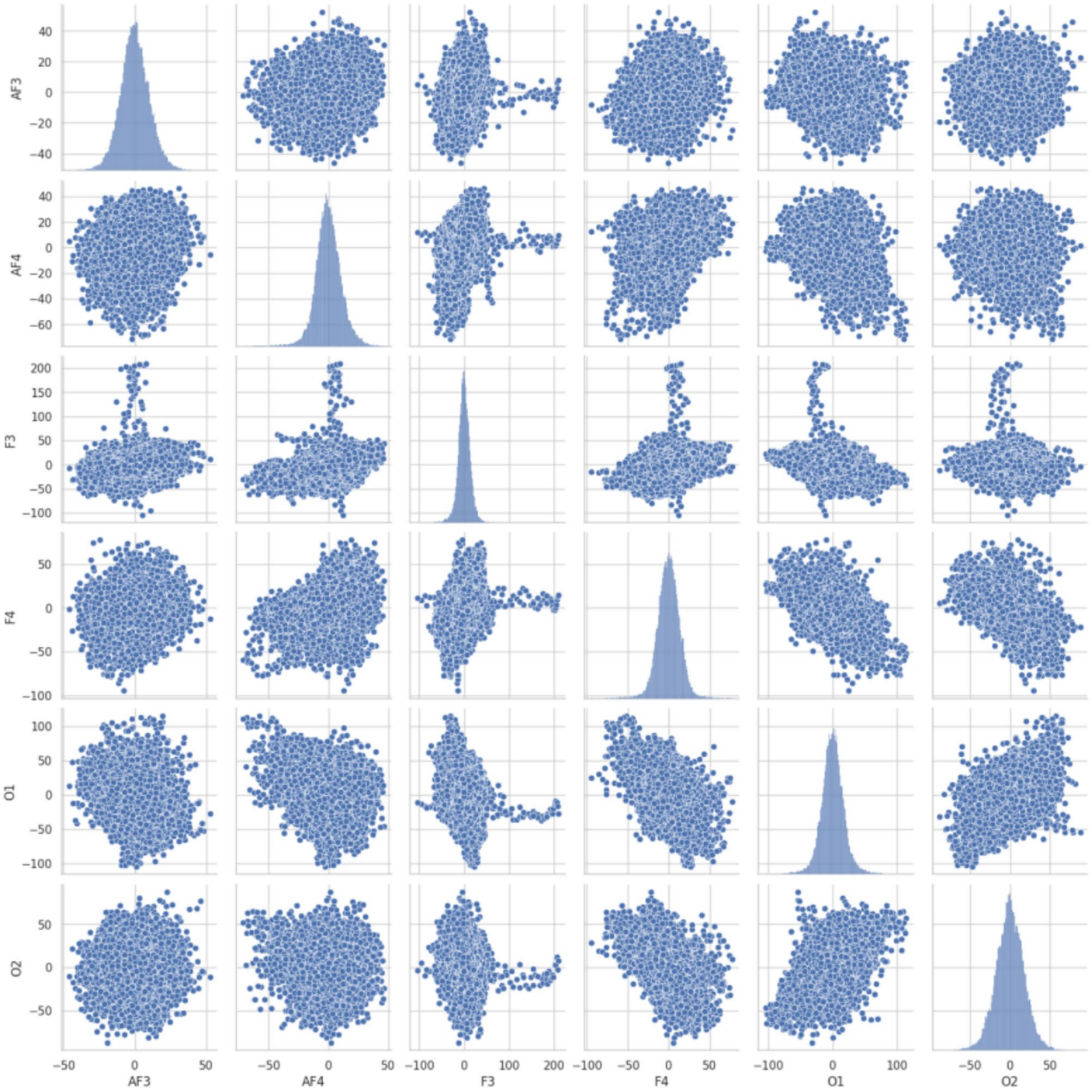
Visualize Relationships between key features.

Figure 14 is a histogram-based distribution of each feature, showing the spread and central tendency of data for each electrode, such as AF3, AF4, etc., with varying ranges, means, and standard deviations across multiple channels,elucidating feature variability and statistical features for model training and exploration of subject generalization. Figure 15 provides a correlation matrix that demonstrates the pair-wise relationships between different featuresacross multiple channels(AF3, AF4, F3, F4, F7, F8, FC5, FC6, O1, O2, P7, P8, T7, and T8). Warmer colors specify strong positive correlations, while cooler colors signify weak or negative correlations. Particularly, frontal channels such as AF3–AF4 and F3–F4 display higher correlation values, signifying shared feature characteristics, while temporal and occipital channels display comparatively lower correlations, representing corresponding information beneficial for emotional state classification. Figure 16 represents the pair-wise relationships between key features across multiple channels represented in a scatterplot matrix. The diagonal plots denote the marginal distributions of single features, representing their central tendency and spread, whereas the off-diagonal scatter plots portray pairwise feature interactions. Numerous feature pairs show clustered and partially linear patterns, signifying correlated behavior, whereas others display widely dispersed distributions, signifying weak or non-linear relationships. These interaction patterns denote both corresponding and redundant feature combinations. Table 11 represents the distribution of each feature.

**Table 11:** Statistical summary of Extracted EEG features.

Feature	Count	Mean	Std	Min	25%	50%	75%	Max
AF3	38,252	0.013611	10.152945	− 46.013900	− 6.483925	− 0.218545	6.445850	52.488800
AF4	38,252	0.006082	11.071779	− 71.382200	− 6.416525	− 0.227875	6.542250	46.643200
F3	38,252	0.004386	14.352935	− 104.268500	− 7.940150	− 0.302535	8.084525	208.565200
F4	38,252	− 0.006251	13.747399	− 94.795000	− 8.376975	0.060477	8.463400	77.857900
F7	38,252	− 0.005188	25.978797	− 234.664500	− 8.812925	− 0.011204	8.705750	234.889100
F8	38,252	− 0.021498	13.743485	− 86.228000	− 9.363550	− 0.045357	9.219000	69.991000
FC5	38,252	− 0.016589	16.210359	− 83.861800	− 10.079800	0.090598	10.104950	159.357800
FC6	38,252	0.015937	14.471805	− 75.213300	− 9.604250	− 0.143080	9.721700	96.310000
O1	38,252	− 0.029628	18.637859	− 104.469600	− 10.621125	− 0.109125	10.361400	114.401500
O2	38,252	0.005387	17.867029	− 87.812700	− 11.378100	− 0.035530	11.516325	87.487600
P7	38,252	0.000706	21.587647	− 143.605700	− 13.007825	− 0.043945	12.806325	185.011600
P8	38,252	0.001598	11.499994	− 66.375800	− 7.178325	− 0.200560	7.072225	55.629000
T7	38,252	0.032812	14.924300	− 70.475300	− 9.210325	− 0.055330	9.186625	114.101300
T8	38,252	− 0.006881	12.084201	− 57.277100	− 7.917775	0.235105	8.148350	56.314400

**Fig. 17:**
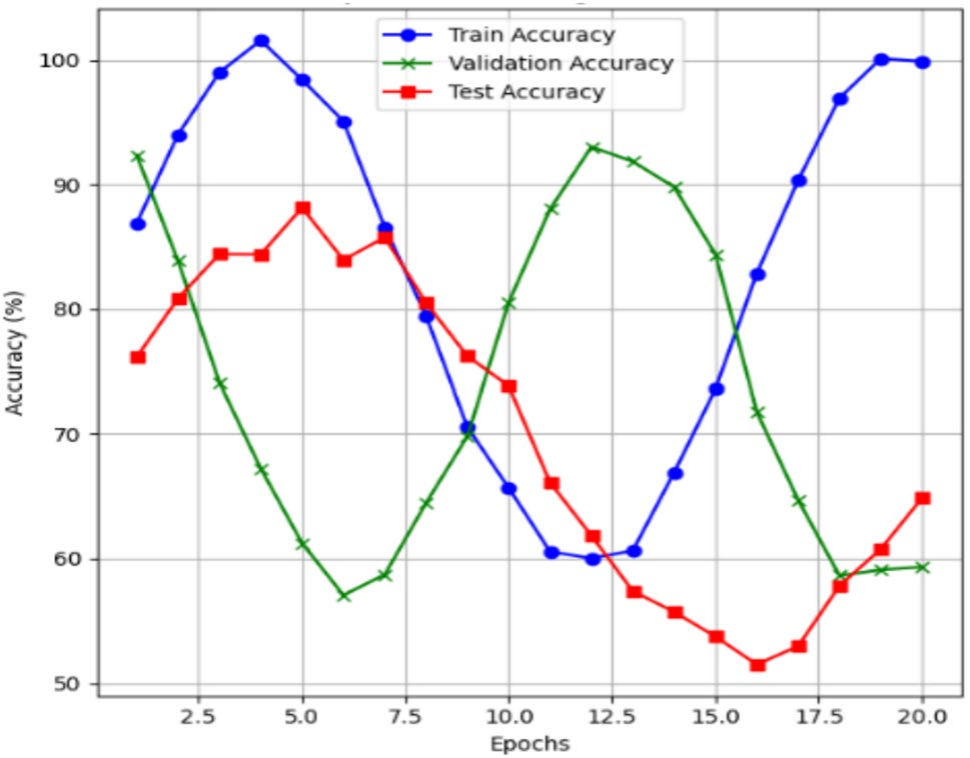
Accuracy of the proposed work.

**Fig. 18:**
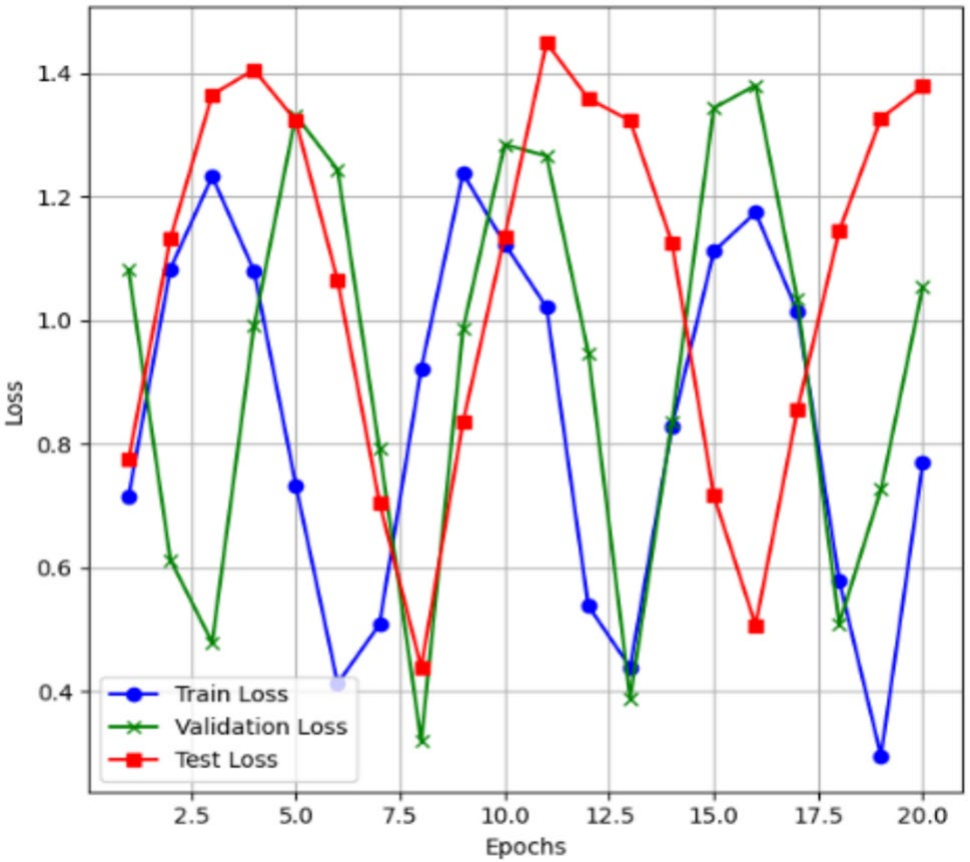
Loss of the proposed work.

Figure 17 showing accuracy curve and Fig. 18 showing the loss curve. From the visualization, the proposed model has high accurate and low loss. Table 12 presents the evaluation metrics of the proposed work with an accuracy of 97.6%, precision of 97.8%, recall of 97.5%, and F1-score of 97.6%, which demonstrates a strong performance. An AUC-ROC value of 0.98 indicates the high discrimination power of the model. Table 13 compares the proposed model with existing deep learning approaches. It can be seen that the proposed method outperforms models such as CNN, LSTM, ResNet50, and VGG16 have higher accuracy and lower loss. It thus highlights the superior efficiency of the proposed work in emotion detection using EEG signals.

**Table 12:** Evaluation metrics of the proposed work.

Metric	Value
Accuracy	97.6%
Precision	97.8%
Recall	97.5%
F1-Score	97.6%
AUC-ROC	0.98

**Table 13:** Accuracy and loss of the proposed Work vs existing deep learning approaches.

Model	Accuracy (%)	Loss
Proposed Work	97.6	0.10
CNN (Convolutional Neural Network)	94.3	0.25
LSTM (Long Short-Term Memory)	95.1	0.18
ResNet50	92.5	0.30
VGG16	93.7	0.22

Model complexity and generalization properties were taken into account in addition to accuracy and loss. The suggested system facilitates effective inference with a single forward pass and has about 3.2 million parameters. The robustness of the suggested method across unseen subjects is further demonstrated by subject-independent evaluation and k-fold cross-validation.

### Discussion

Table 14 depicts the performance of the proposed model on various datasets with a great accuracy of 98.5% and loss of 0.02 on the EEG Brainwave Dataset, 94.5% accuracy with loss of 0.55 on the DEAP Dataset, and 97.6% accuracy with loss of 0.10 on the EDA on Emotion Recognition (S01G1AllChannels) dataset.

**Table 14:** Accuracy and loss of the proposed work.

S. No	Dataset	Accuracy (%)	Loss
1	EEG Brainwave Dataset	98.5	0.02
2	DEAP Dataset	94.5	0.55
3	EDA on Emotion Recognition (S01G1AllChannels)	97.6	0.10

Table 15 compares the proposed work with other recent EEG signal classification models. The proposed model outperforms many of the state-of-the-art approaches, including Cai et al. (2024) at 91.2% accuracy, Khan et al. (2024) at 92.3%, and Liu et al. (2023) at 96.4%. Importantly, the proposed work achieves the highest accuracy on the EEG Brainwave Dataset (98.5%) and performs competitively on the DEAP and EDA datasets, thus showing the robustness of the model across multiple EEG datasets. In order to diminish the overfitting issues that occurs due to the complexity of the model and inadequate data, multiple regularization approaches were implemented. These comprises of dropout layers within the GNN and MANN modules, L2 weight regularization, and early stopping with respect to validation loss convergence. In order to prevent the model from issues such as data leakage, overfitting, the training was performed from same subjects such that the same samples did not reflect in both training and test splits. Furthermore, k-fold cross-validation was implemented to improve the consistency of the model’s performance across several subjects. These measures pave way for improving generalization and overfitting issues, despite the huge size of the model’s parameters

**Table 15:** Comparative performance analysis of EEG Signal classification models.

S. No	Author et al. (Year)	Method	Accuracy (%)	Loss
	Cai et al. (2024)	Spectral Image via EEG-SWTNs	91.2	0.34
	Khan et al. (2024)	Convolutional Networks (CNN)	92.3	0.30
	Tan et al. (2024)	EEG Signals (S3T-Net)	93.1	0.28
	Akhand et al. (2024)	Feature Map Enhancement (Mutual Info)	94.2	0.26
	Liu et al. (2023)	CNN + Capsule Network (Attention)	96.4	0.18
	Gunda et al. (2024)	Lightweight Attention (CNN + Attention)	95.7	0.22
	Lin et al. (2023)	Improved Graph Neural Network (GNN)	94.8	0.20
	Proposed Work	EEG Brainwave Dataset	98.5	0.02
	Proposed Work	DEAP Dataset	94.5	0.55
	Proposed Work	EDA on Emotion Recognition (S01G1AllChannels)	97.6	0.10

Table 16 presents an ablation study of the proposed method, evaluating the contribution of various components to the performance. The full model comprising GNN + MANNs, MSWT, Kalman filtering, and Spectral Entropy attained 97.6% accuracy with a loss of 0.10, precision of 97.8%, recall of 97.5%, F1-score of 97.6%, and AUC-ROC of 0.98. All reported performance metrics correspond to the average values obtained across k-fold cross-validation.

**Table 16:** Ablation study of the proposed method.

S.No	Experiment configuration	Accuracy (%)	Loss	Precision (%)	Recall (%)	F1-Score (%)	AUC-ROC
1	Full Model (with GNN + MANNs, MSWT, Kalman filtering, and Spectral Entropy)	**97.6**	**0.10**	**97.8**	**97.5**	**97.6**	**0.98**
2	Without GNN (MANNs, MSWT, Kalman filtering, Spectral Entropy)	95.2	0.12	95.6	94.3	94.9	0.95
3	Without MANNs (GNN, MSWT, Kalman filtering, Spectral Entropy)	94.1	0.14	94.0	92.8	93.4	0.94
4	Without MSWT (GNN + MANNs, Kalman filtering, Spectral Entropy)	93.7	0.16	93.5	92.2	93.0	0.93
5	Without Kalman filtering (GNN + MANNs, MSWT, Spectral Entropy)	92.5	0.18	92.3	91.1	91.7	0.92
6	Without Spectral Entropy (GNN + MANNs, MSWT, Kalman filtering)	91.2	0.20	91.0	89.9	90.4	0.91
7	Without MSWT & Kalman filtering (GNN + MANNs, Spectral Entropy)	90.3	0.22	90.1	88.7	89.4	0.90
8	Without GNN & MANNs (MSWT, Kalman filtering, Spectral Entropy)	88.6	0.25	88.2	86.4	87.2	0.88

Significant values are in bold.

Removal of any particular component resulted in decreased performance. For example, the major performance deprivation occurs when GNN is removed, the corresponding accuracy decreased by 95.2%, representing the important aspects of spatial modeling in emotion recognition. Emotional evidence in EEG signals is dispersed among interacting brain sections than remote channels; therefore, openly modelling inter-channel relationships is necessary. The GNN module seizures the spatial dependencies by learning relational representations between electrodes, allowing the model to exploit functional connectivity patterns connected with emotional states.

Similarly, excluding MANN resulted in further lower accuracy with only 94.1%. This reduction advises that the MANN principally extends temporal consistency and stability by conserving long-term contextual information among emotional epochs. While temporal modelling progresses robustness, it is lesser to accurate spatial representation in this task. The entire model welfares from the balancing interaction of spatial connectivity (GNN) and long-term temporal memory (MANN), elucidating the greater performance perceived in the proposed framework.Meanwhile, removing MSWT results in an accuracy of 93.7%, and omitting Kalman filtering reduces accuracy to 92.5%. The model performs highly when Spectral Entropy is removed with a performance loss of 91.2% and worse when MSWT and Kalman filtering are excluded with a performance loss of 90.3%. Furthermore, Table 17 highlights the functional role and neurophysiological significance of the foremost component of the proposed framework.

**Table 17:** Interpretability analysis of the proposed model components.

S.No	Model component	Learning mechanism	Interpretability and Neurophysiological Insight
1	Channel selection – Reinforcement learning	Highlights frontal and prefrontal EEG channels among subjects and emotional states	Frontal and prefrontal regions are powerfully connected with emotional states and affective processing, signifying that the learned channel significance supports the neurophysiological findings
2	GNN	Biased inter-channel connections signifying spatial dependencies between electrodes	Seizures functional connectivity patterns scattered across different reins of brain, imitating coordinated neural activity tangled in emotional processing
3	MANN	Preserves noticeable temporal representations between emotional epochs	Captures long-term contextual information, enhancing stability of emotion depiction with respect to temporal and subject variability
4	Temporal Attention Mechanism	Highlights emotionally noticeable EEG segments during classification	Captures serious temporal windows related with emotional states with robust temporal modeling
5	Multi-scale wavelet features	Translates frequency-specific data across several resolutions	Preserves the known emotional correlations in EEG, where different frequency bands contribute differently to emotional states

The suggested framework is meant to support established findings in affective neuroscience as well as categorization performance. Frontal cortical activity and frequency-specific oscillations, especially in the alpha, beta, and gamma bands, are recognized to be indicators of emotional processes in EEG. By decomposing EEG data across several temporal and spectral resolutions, the multi-scale wavelet transform (MSWT) makes it possible to extract features that represent both transient and sustained emotional dynamics. This representation is in line with earlier research that connected band-specific brain oscillations to emotional valence and arousal.

By increasing signal stability while maintaining the inherent temporal structure of EEG activity, Kalman filtering further strengthens the framework. The adaptive nature of Kalman filtering lowers measurement noise and non-stationary artifacts instead of suppressing emotion-related changes, making it possible to recover emotion-relevant characteristics more reliably. Furthermore, research on frontal asymmetry and widespread cortical participation in affective processing is consistent with the focus on frontal and prefrontal channels through adaptive channel selection. When combined, these elements guarantee that the suggested pipeline stays based on neurophysiological principles while tackling real-world issues in EEG-based emotion identification.

## Conclusion

The proposed emotion recognition system provides outstanding progress in the processing and analysis of EEG signals to achieve dynamic emotion detection. The method offers quality preprocessing of signals with strong elimination of noise and keeping relevant features by combining multi-scale wavelet transform and kalman filtering with wavelet denoising. The added analysis of spectral entropy improves the technique by allowing a clear indication of emotional peaks with further segmentation of the EEG signal into meaningful epochs. Dynamic optimization of channel selection using reinforcement-based deep Q-Networks will improve the adaptability of the system by changing emotional states and reducing computational complexity. In the feature extraction phase, a hybrid Spatio-Temporal Attention Network(ST-AN) was used to uncover spatial and temporal patterns in EEG data, while Multi-Scale Feature Fusion captured both short- and long-term dependencies. The classification is strong and robust, as it is performed by an ensemble of Graph Neural Networks(GNN) and Memory-Augmented Neural Networks (MANN). Therefore, the proposed system ensures adaptability for different subjects and emotional states. On the EEG Brainwave Dataset, it achieves 98.5% accuracy and 0.02 loss. On the DEAP Dataset, it obtains an accuracy of 94.5% with a loss of 0.55. The model reaches 97.6% accuracy with a loss of 0.10 for the EDA on Emotion Recognition.

## Data Availability

The datasets used and/or analysed during the current study available from the corresponding author on reasonable request.
